# Investigating the molecular mechanisms underlying the anti‐CRISPR function of AcrIIA13b protein

**DOI:** 10.1111/febs.70304

**Published:** 2025-10-24

**Authors:** So Yeon Lee, Hyun Ho Park

**Affiliations:** ^1^ College of Pharmacy Chung‐Ang University Seoul Korea; ^2^ Department of Global Innovative Drugs Graduate School of Chung‐Ang University Seoul Korea

**Keywords:** AcrIIA13b, adaptive immunity, anti‐CRISPR, CRISPR‐Cas system, crystal structure

## Abstract

The CRISPR‐Cas systems of adaptive immunity in bacteria and archaea provide resistance against phages and other mobile genetic elements. Counteractive anti‐CRISPR (Acr) proteins in phages and archaeal viruses impede these CRISPR‐Cas systems. Although CRISPR‐Cas systems have revolutionized genome editing, potential off‐target events remain a safety concern. Hence, a thorough comprehension of the structural and molecular basis of diverse Acrs is imperative to unravel the fundamental mechanisms governing CRISPR‐Cas regulation. Here, we present the structure of AcrIIA13b from *Staphylococcus haemolyticus* and analyze its structural and functional features to reveal the molecular basis underlying the inhibition of Cas9 by AcrIIA13b. Our structural analysis shows that AcrIIA13b eliminates the cleavage activity of *Staphylococcus aureus* Cas9 (SauCas9) by blocking the PAM‐binding region of Cas9 so that Cas9 cannot recognize the target DNA. In addition, we demonstrate that the 15 amino acid residues at the N terminus of AcrIIA13b, which were revealed to be important for its dimerization, are critical for its inhibitory activity against Cas9. Our findings shed light on the molecular basis of AcrIIA13b‐mediated CRISPR‐Cas inhibition and provide valuable insights into the arms race between bacteria and phages.

## Graphical abstract

Anti‐CRISPR protein AcrIIA13b inhibits CRISPR–Cas9 function by preventing target DNA engagement. The Cas9–sgRNA ribonucleoprotein (RNP) normally recognizes the protospacer adjacent motif (PAM) duplex to initiate DNA cleavage. AcrIIA13b binding to the PAM‐interacting WED–PI domain blocks this recognition, leaving the target DNA intact. This structural mechanism explains how AcrIIA13b suppresses Cas9‐mediated genome interference and highlights its role as a molecular inhibitor of CRISPR–Cas9 activity.

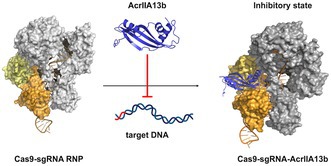

**Abbreviations**
Acr: anti‐CRISPR
ASU: asymmetric unit
Cas: CRISPR‐associated
CD: circular dichroism
CRISPR: clustered regularly interspaced short palindromic repeats
crRNA: CRISPR RNA
CSS: complexation significance score
HNH: histidine(H)‐asparagine(N)‐histidine(H) motif
HTH: helix‐turn‐helix
ITC: isothermal titration calorimetry
MALS: multi‐angle light scattering
MolA: molecule A
MR: molecular replacement
Ni‐NTA: nickel‐nitrilotriacetic acid
PAM: protospacer adjacent motif
PI: PAM‐interacting domain
PPI: protein–protein interface
REC: recognition domain
RMSD: root mean square deviation
RNP: ribonucleoprotein
RuvC: crossover junction endodeoxyribonuclease
SauCas9: *Staphylococcus aureus* Cas9
SDS/PAGE: sodium dodecyl sulfate/polyacrylamide gel electrophoresis
SEC: size‐exclusion chromatography
sgRNA: single‐guide RNA
shAcrIIA13b: *Staphylococcus haemolyticus* AcrIIA13b
SymA: symmetry A
WED: wedge‐like domain
ΔN15: N‐terminal 15 residue deletion

## Introduction

As a result of the long evolutionary battle for survival, bacteria have evolved a sophisticated adaptive immune system called the Clustered Regularly Interspaced Short Palindromic Repeats (CRISPRs) and CRISPR‐associated proteins (Cas). CRISPR‐Cas systems store genetic information from past infections, allowing bacteria to recognize and degrade foreign DNA upon reinfection. This bacterial immune response occurs in a two‐stage process involving expression and interference, where processed CRISPR RNAs (crRNAs) guide Cas proteins to cleave foreign DNA [1, 2, 3, 4, 5]. Because of its site‐specific cleavage, the CRISPR‐Cas system has been widely adapted for gene editing applications to modify specific genes for experimental and medicinal purposes [6, 7].

CRISPR‐Cas systems are classified into two classes (Class 1 and Class 2), encompassing seven types (types I–VII), based on their genetic composition and mechanism. Class 1 systems (types I, III, IV, and VII) use multi‐subunit effectors, while Class 2 systems (types II, V, and VI) rely on single‐protein effectors [8, 9, 10]. Among them, Type II‐Cas9 systems are the most widely studied due to their simplicity and efficiency. Cas9 recognizes a protospacer adjacent motif (PAM), undergoes a conformational change upon DNA binding, and activates cleavage through HNH and RuvC nuclease domains [11, 12, 13, 14]. This simplicity and accuracy have made CRISPR‐Cas9 a powerful tool in genome‐editing technology [15, 16, 17].

To survive the battle for persistence, phages can escape from bacterial immunity by encoding anti‐CRISPR proteins (Acrs) that block the CRISPR‐Cas systems [18, 19, 20]. These Acrs exhibit diverse mechanisms of inhibition, RNA sequestration, competitive blocking, DNA‐binding domains, and blocking nuclease domains. Acr families are typically named based on the type of CRISPR‐Cas system they inhibit, for example, AcrIF for Type I‐F and AcrIIA for Type II‐A systems [20, 21, 22].

AcrIIAs have been found in diverse bacterial species, suggesting their widespread role in regulating CRISPR‐Cas activity. Interestingly, when phage‐encoded Acr genes integrate into the bacterial genome, the host can become a reservoir of Acr proteins, suppressing CRISPR‐Cas immunity and facilitating reinfection by phages lacking their own Acr genes [23]. Previous studies on AcrIIA28, AcrIIA4, and AcrIIA14 have provided valuable insights into the inhibitory mechanisms employed by this family, revealing interactions directly with Cas9 [24, 25, 26, 27, 28, 29, 30, 31, 32].

**Fig. 1:**
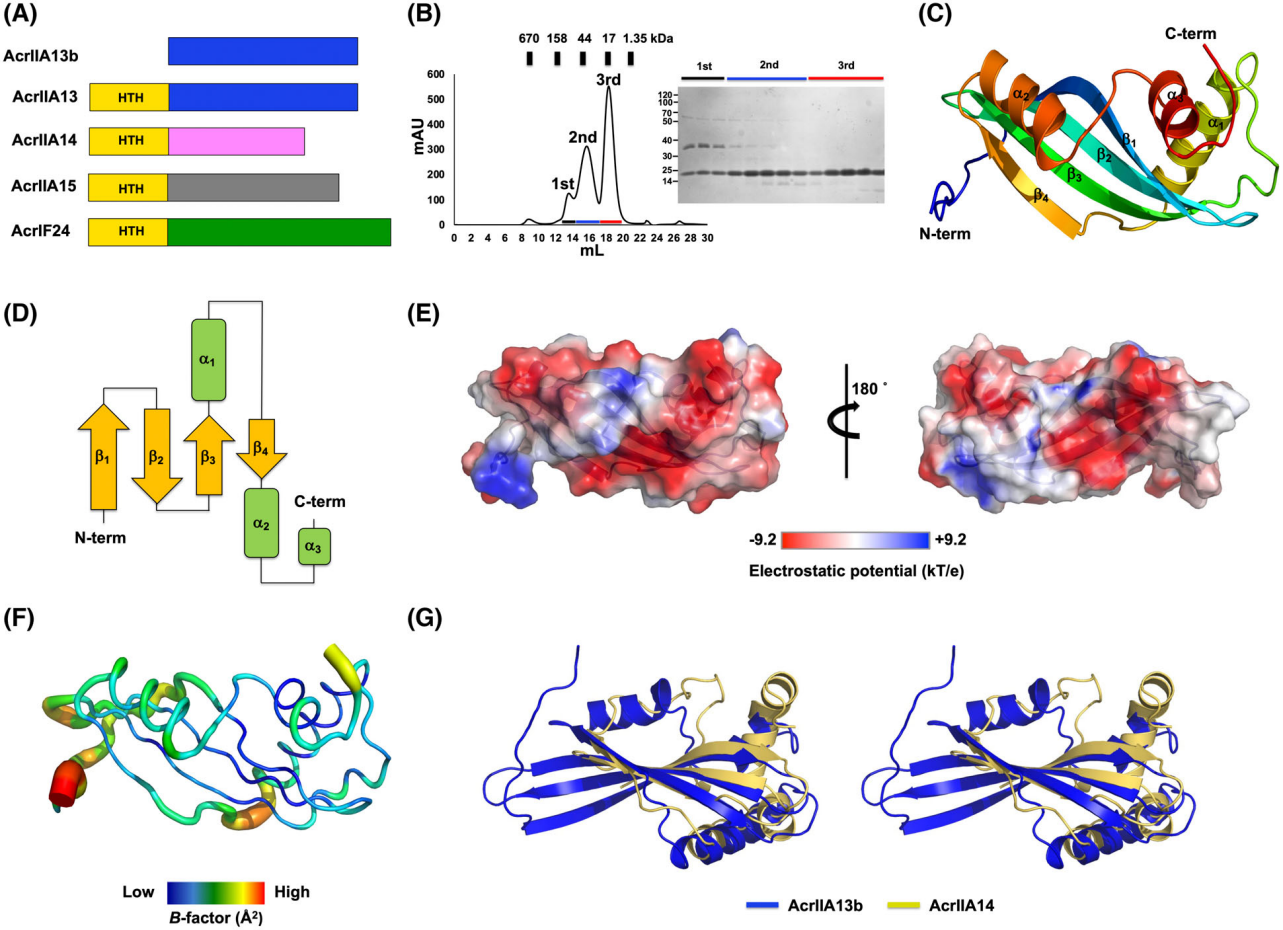
The crystal structure of AcrIIA13b reveals a novel feature that contains an acidic surface. (A) Helix‐turn‐helix (HTH) domain‐containing Acrs and domain comparison with AcrIIA13b. (B) Size‐exclusion chromatography (SEC) profile of AcrIIA13b. A sodium dodecyl sulfate/polyacrylamide gel electrophoresis (SDS/PAGE) gel of the three peak fractions is shown on the right of the profile. Fractions corresponding to each peak are indicated by horizontal bars: black (1st peak), blue (2nd peak), and red (3rd peak). These fractions were collected and analyzed by SDS/PAGE, with lane colors matching the bar colors on the SEC profile. Data shown are representative of three independent experiments (*n* = 3). (C) Cartoon representation of AcrIIA13b. The color of the chain from the N to the C terminus gradually moves through the spectrum from blue to red. The three α‐helices and four β‐sheets are labeled α_1_–α_3_ and β_1_–β_4_, respectively. Structural visualization was performed using pymol. (D) Topology representation of AcrIIA13b. (E) Surface electrostatic potential of AcrIIA13b. The respective surface electrostatic distributions are represented by a scale ranging from −9.2 kT/e (red) to +9.2 kT/e (blue). Generated using pymol. (F) *B*‐factor distribution in the structure of AcrIIA13b. The structure is presented in a putty representation using pymol. Rainbow colors from violet to red with increasing *B*‐factor values were used for *B*‐factor visualization. (G) Structural superimposition of AcrIIA13b (blue) with AcrIIA14 (gold) using pymol (Schrödinger, New York, NY, USA), which is the most structurally similar protein to AcrIIA13b. Stereo view was provided for better visualization.

AcrIIA13, identified in *Staphylococcus* species, is a potent and selective inhibitor of *Staphylococcus aureus* Cas9 (*Sau*Cas9) [33]. AcrIIA13 exerts the most robust inhibition of the *Sau*Cas9 activity compared with AcrIIA14 and AcrIIA15. While AcrIIA13, AcrIIA14, and AcrIIA15 share an N‐terminal helix‐turn‐helix (HTH) domain associated with transcriptional regulation, the truncated variant AcrIIA13b lacks this HTH domain and was found in *Staphylococcus haemolyticus* (Fig. 1A) [33].

In this study, we present the high‐resolution (1.53 Å) crystal structure of AcrIIA13b from *St. haemolyticus* and analyze its structural and functional features to elucidate the molecular basis of Cas9 inhibition. We show that AcrIIA13b abolishes Cas9 cleavage activity by directly occupying the PAM‐binding region, thereby blocking target DNA recognition. Furthermore, the N‐terminal 15 residues contribute to dimerization and are important for inhibitory function, although they are not required for Cas9 binding. Notably, AcrIIA13b exhibits potent inhibition regardless of the order of addition, including under pre‐cleavage conditions where R‐loop formation is likely initiated. These features suggest that AcrIIA13b represents a structurally and functionally distinct variant within the AcrIIA family.

Our findings shed light on the molecular basis of AcrIIA13b‐mediated Cas9 inhibition and provide valuable insights into the arms race between bacteria and phages. The structural information presented here might be helpful in the development of strategies to modulate CRISPR‐Cas activity for precise genome editing and control of gene expression. Moreover, a comprehensive understanding of the AcrIIA family's structure and function will deepen the knowledge of bacterial defense strategies and pave the way for the discovery of novel anti‐CRISPR mechanisms.

## Results

### The crystal structure of AcrIIA13b from *St. haemolyticus* reveals a novel feature that contains an acidic surface

The inhibitory mechanism of AcrIIA13b on Cas9 was investigated by overexpressing and purifying full‐length AcrIIA13b for structural analysis. The purification process involved Ni‐NTA affinity chromatography and SEC. Three distinct peaks were observed during SEC, and SDS/PAGE analysis confirmed that all three peaks contained AcrIIA13b protein, although numerous impurities were present, especially in the 1st peak. The 2nd and 3rd peaks eluting at around 16 and 18 mL contained the purest AcrIIA13b protein, as judged by SDS/PAGE (Fig. 1B). We initially tried to make a protein crystal using the two protein samples eluted in the 2nd peak and 3rd peak. Among them, the protein of interest eluting at approximately 18 mL (3rd peak) was successfully crystallized, and diffraction data were collected at a resolution of 1.53 Å at the PAL synchrotron.

MR phasing was performed using a predicted structural model from alphafold2 [34], leading to the final structural model of AcrIIA13b. The final model was refined to *R*_work_ = 21.43% and *R*_free_ = 23.71%. The crystal belonged to space group *C2* and contained one molecule in the asymmetric unit (ASU) (Fig. 1C). The refinement statistics are summarized at Table 1. The final structural model encompassed most of AcrIIA13b (residues from K7 to E129), except for the first six amino acids at the N terminus, which had unclear electron density and were excluded from the model (Fig. 1C). The AcrIIA13b structure comprised four β‐strands (β1–β4) and three α‐helices (α1–α3) (Fig. 1D). Three β‐strands (β1–β3) were located at the N terminus, with one α‐helix (α1) connecting β3 and β4, and α2 and α3 situated at the C‐terminus (Fig. 1D). Analysis of the surface electrostatic potential showed that negative charges were predominantly distributed along the β‐strands (Fig. 1E). This acidic surface characteristic may suggest a potential for AcrIIA13b to engage with positively charged regions of Cas9, such as DNA/RNA binding domains. *B*‐factor analysis revealed high rigidity with low *B*‐factor values (average 27.53 Å) for most of the structure, except for the N‐terminal region (Fig. 1F), which may reflect its role in mediating functional interactions.

**Table 1:** Data collection and refinement statistics.

Data collection	
Space group	*C 1 2 1*
Unit cell parameter	
*a*, *b*, *c* (Å)	*a* = 102.51, *b* = 37.47, *c* = 37.09
α, β, γ (°)	α = 90, β = 103.288, γ = 90
Resolution range (Å)^a^	26.24–1.53
Total reflections	141 233
Unique reflections	19 723
Multiplicity^a^	7.2 (7.4)
Completeness (%)^a^	94.19 (93.55)
Mean *I*/σ(*I*)^a^	18.82 (1.53)
*R*_merge_ (%)^a^, ^b^	4.4 (132.9)
*R*_meas_ (%)^a^	4.7 (142.7)
CC1/2^a^	1 (0.73)
Wilson *B*‐factor (Å^2^)	27.53
Refinement	
Resolution range (Å)	26.24–1.53
Reflections	19 704
*R*_work_ (%)	21.4
*R*_free_ (%)	23.71
No. of molecules in the asymmetric unit	1
No. of non‐hydrogen atoms	1114
Macromolecules	1029
Solvent	85
Average *B*‐factor values (Å^2^)	33.02
Macromolecules	32.45
Solvent	39.31
Ramachandran plot: favored/allowed/outliers (%)	98.36/1.64/0.00
Rotamer outliers (%)	0
Clashscore	1.3
RMSD bonds (Å)/angles (°)	0.008/0.99

^a^ Values for the outermost resolution shell in parentheses.

^b^ *R*_merge_ = ∑_*h*_∑_*i*_ *|I*(*h*)_*i*_ − <*I*(*h*)>|/∑_*h*_∑_*i*_I(*h*)_*i*_, where *I*(*h*) is the observed intensity of reflection *h*, and <*I*(*h*)> is the average intensity obtained from multiple measurements.

To explore proteins with similar structures, we used the Dali server [35], which identified AcrIIA14 as the most structurally similar protein (Table S1). Although AcrIIA14 was picked as the closest related structure, the *Z*‐score (5.7) and sequence identity (13%) and RMSD value (2.7 Å) are too low to be a structurally similar protein. Upon aligning the structures of AcrIIA13b and AcrIIA14, it was observed that certain parts of their structures, including the β‐sheet (β1–β3) and α‐helices (α1 and α3), shared similar folding patterns. This resemblance may imply a potential for direct Cas9 binding, as seen in AcrIIA14, which is known to inhibit Cas9 by targeting its HNH domain. Despite this general similarity, AcrIIA13b also exhibits distinct structural features, including additional α2 helices and elongated β‐strands not present in AcrIIA14 (Fig. 1G). These novel features suggest that AcrIIA13b may utilize a previously uncharacterized structural framework, potentially supporting a different mode of Cas9 inhibition. Together, these observations indicate that while AcrIIA13b shares a common structural core with AcrIIA14, it also harbors unique architectural features that may contribute to its distinct inhibitory mechanism.

### AcrIIA13b forms a dimer via its N‐terminal beta‐strand in solution

**Fig. 2:**
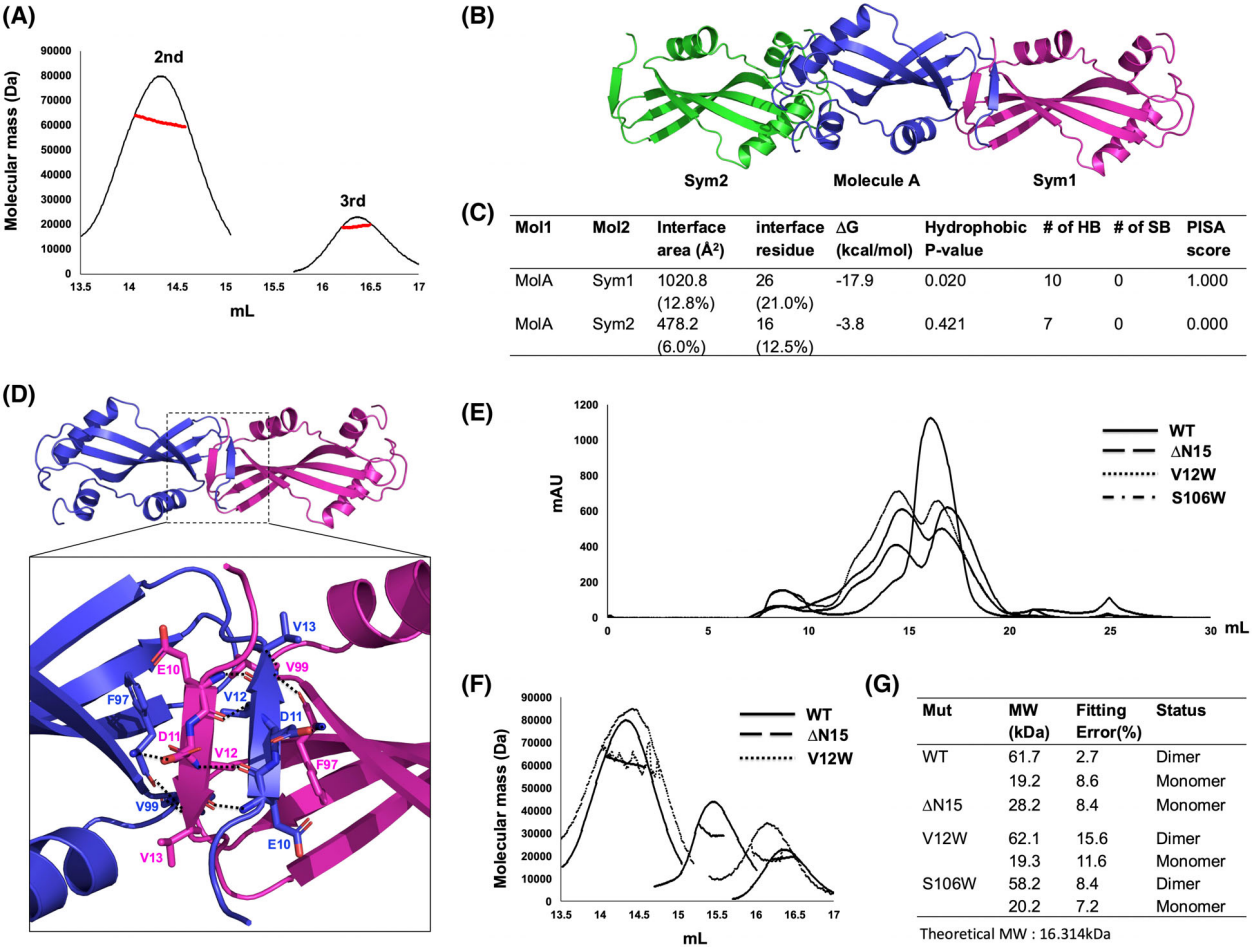
AcrIIA13b forms a dimer via its N‐terminal loop in solution. (A) Size‐exclusion chromatography (SEC)–multi‐angle light scattering (MALS) profile of AcrIIA13b. In the MALS profile, the experimental MALS data (red line) are plotted as SEC elution volume (*x*‐axis) versus absolute molecular mass (*y*‐axis) distributions on the SEC chromatogram (black) at 280 nm. Data are representative of three independent experiments (*n* = 3). (B) Crystallographic packing symmetry analysis using pymol. A single molecule found in the asymmetric unit (ASU) is indicated by the blue‐colored ribbon structure, and the two symmetry molecules found by packing analysis are indicated by magenta‐colored (Sym1) and green‐colored (Sym2) ribbon structures. (C) Table summarizing the interaction details of the two types of putative interfaces analyzed by the PISA server. *n* = 1, as this is a computational prediction based on one crystal structure. (D) Putative dimeric structure of AcrIIA13b generated, analyzed by crystal packing and the PISA server and visualized in pymol. The region of protein–protein interactions (PPI) magnified and presented in the lower panel is indicated by a black‐dashed square. A close‐up view of PPI in the dimeric structure of AcrIIA13b is provided in the lower panel. The black‐dashed lines indicate hydrogen bonds. *n* = 1, as this structure is generated from a single crystallographic dataset and computational interface prediction. (E and F) Validation of the PPI via mutagenesis. SEC (E) and MALS (F) profiles comparing the position of eluted peaks of various mutants with wild‐type. Gray lines in the peaks in panel F indicate the experimental molecular mass measured by MALS. Representative of three experiments (*n* = 3). (G) Table summarizing the result of SEC–MALS. Mut and MW indicate mutant and molecular weight, respectively. Fitting error indicates the MALS fitting error. The theoretical molecular weight is indicated below the table.

Although many Acrs inhibit the activity of Cas9 in monomeric form, it was previously established that some Acrs function in dimeric form [32, 36, 37, 38]. For AcrIIA13b, the SEC results showed three main peaks for the target (Figs 1B and 2A), suggesting the coexistence of the dimeric, monomeric, and even higher oligomeric forms in solution. To determine the stoichiometry of AcrIIA13b, MALS was employed. Results from the MALS analysis indicated an experimental molecular mass of 61.78 kDa (2.72% fitting error) for the 2nd peak and 19.23 kDa (8.67% fitting error) for the 3rd peak (Fig. 2A). Given the theoretical molecular mass of monomeric AcrIIA13b with a C‐terminal 6 × His‐tag (16.3 kDa), the 3rd peak corresponds well to the monomeric form. Although the 2nd peak's molecular mass is slightly higher than expected for a dimer, we concluded it likely represents the dimeric form of AcrIIA13b. This overestimation may stem from the elongated, beta‐strand mediated dimer conformation, which could affect hydrodynamic properties or detector response in SEC‐MALS analysis (Fig. 2B). Taken together with the SEC profile and structural insights, these results support the interpretation that the 2nd peak corresponds to the dimeric form of AcrIIA13b.

Further crystallographic packing analysis revealed two types of putative dimers: a MolA/Sym1 dimer and a MolA/Sym2 dimer (Fig. 2B). The MolA/Sym1 dimer was formed through the N‐terminal beta‐strand (residue K7–G15) of each molecule, while the MolA/Sym2 dimer involved helix α1 from one molecule and helix α3 of another molecule. To identify the biologically relevant form, protein–protein interactions (PPI) were analyzed using the PDBePISA server [39]. The PPI analysis showed that the MolA/Sym1 dimer had a complexation significance score (CSS) of 1.0, indicating its greater biological relevance compared to the MolA/Sym2 dimer with a score of 0 (Fig. 2C). A total of 52 residues (26 residues from each molecule) were involved in the PPI of the MolA/Sym1 dimer, whose total surface buried an area of 1020.8 Å^2^, representing 12.8% of the total surface area (Fig. 2C). The MolA/Sym1 dimer interface was maintained by 10 hydrogen bonds, mainly formed by the N terminus beta‐strand from both molecules (Fig. 2D). Meanwhile, 32 residues (16 residues from each molecule) were involved in the formation of the MolA/Sym2 dimer, whose total surface buried an area of 478.2 Å^2^, representing only 6.0% of the total surface area (Fig. 2C). Based on this preliminary PPI analysis, we propose that AcrIIA13b naturally forms a MolA/Sym1 dimer in solution.

To experimentally confirm the relevance of the MolA/Sym1 dimer, AcrIIA13b mutants were generated and examined for dimer disruption. For this purpose, we generated two tentative MolA/Sym1 PPI disruption mutants, V12W and ΔN15 (deletion of the first 15 N‐terminal amino acid residues), and one tentative MolA/Sym2 PPI disruption mutant, S106W. The V12W mutant, which could disrupt four hydrogen bonds, did not affect the dimer formation, whereas the ΔN15 mutant, predicted to eliminate all possibilities of forming hydrogen bonds, showed a delay of the oligomeric peak (2nd peak) in SEC, indicating disrupted dimer formation (Fig. 2E). Like the V12W mutant, the S106W mutant also did not affect the SEC profile, indicating that S106W failed to disrupt the MolA/Sym2 PPI. Besides the SEC profile comparison, the absolute molecular weight of the mutants was further examined by MALS, and the results supported the disruption observed in the SEC analysis. The V12W mutant showed molecular weights of 62.1 kDa (15.6% fitting error) and 19.3 kDa (11.6% fitting error), similar to the wild‐type. In contrast, the ΔN15 mutant exhibited a calculated molecular weight of 28.2 kDa (8.4% fitting error), which is notably reduced compared to the wild‐type dimer (~ 61.7 kDa), indicating the disruption of dimerization. Although this value is closer to a dimer than a strict monomer, the shift in SEC elution volume and the significantly decreased apparent molecular weight collectively support a predominantly monomeric form (Fig. 2F,G). Based on comprehensive structural analysis, crystal packing examination, and mutagenesis studies, followed by the dimer disruption assay, it was concluded that AcrIIA13b exists as a symmetric MolA/Sym1 dimer in solution.

### AcrIIA13b inhibits the cleavage activity of Cas9, regardless of order of addition of reaction components

**Fig. 3:**
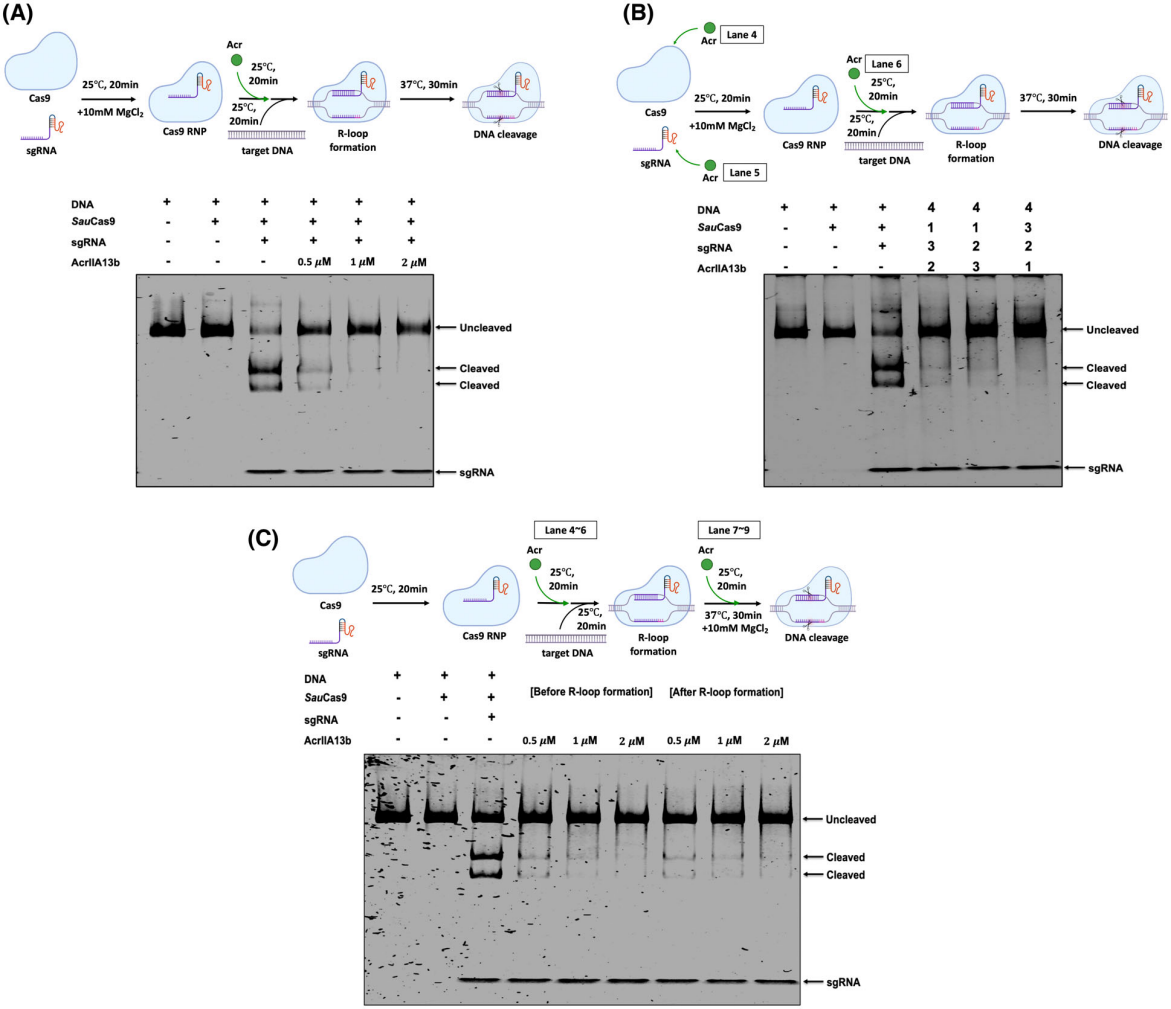
AcrIIA13b inhibits the cleavage activity of Cas9, regardless of order of addition of reaction components. (A and B) *In vitro* anti‐CRISPR activity assay was performed on 4% polyacrylamide gels that were stained with SYBR Gold. Amount of AcrIIA13b added in the reaction indicated in (A). The numbers in (B) indicate the order of components added to the enzyme reaction. ‘1’ represents the first sample added, and ‘4’ represents the last sample added. ‘+’ and ‘−’ indicate added and not added to the enzyme reaction, respectively. (C) *In vitro* anti‐CRISPR activity assay to determine whether AcrIIA13b operates before or after R‐loop formation of ribonucleoprotein (RNP) complex. The sample for ‘Before R‐loop formation’ was produced by incubating Cas9^+^ sgRNA^+^ AcrIIA13 mixture first, and then adding DNA and Mg^2+^, while ‘After R‐loop formation’ sample was produced by incubating Cas9^+^ sgRNA^+^ DNA mixture first, and then adding AcrIIA13b and Mg^2+^. ‘+’ and ‘–’ indicate added and not added to the enzyme reaction, respectively. To help visualize the experimental setup, schematic illustrations above each gel panel depict the order of addition of Cas9, sgRNA, AcrIIA13b, and target DNA. The timing of AcrIIA13b addition is specifically indicated with green arrows. Each illustrated condition corresponds to the lane numbers indicated in the gels. Data representative of three independent experiments (*n* = 3).

In order to test the inhibitory activity of AcrIIA13b on Cas9, we conducted an *in vitro* DNA cleavage assay using SauCas9 and an 1100 bp double‐stranded DNA (dsDNA) substrate containing the target sequence with a 5′‐NNGRRT‐3′ PAM. A specific sgRNA was used to guide Cas9 to the target site. As expected, Cas9 efficiently cleaved the target DNA only when pre‐incubated with sgRNA to form the ribonucleoprotein (RNP) complex (Fig. 3A). Upon addition of increasing concentrations of AcrIIA13b, DNA cleavage was progressively reduced, and at 2 μm AcrIIA13b, cleavage activity was nearly abolished, with the cleaved DNA fragment no longer visible on the gel (Fig. 3A). These results demonstrate that AcrIIA13b potently inhibits SauCas9‐mediated DNA cleavage in a concentration‐dependent manner.

To further analyze whether the inhibitory activity of AcrIIA13b depends on the order of component assembly, we conducted *in vitro* cleavage assays in which the sequence of Cas9, sgRNA and AcrIIA13b addition was systematically varied prior to initiating DNA cleavage. All other experimental conditions were maintained consistently. Remarkably, efficient inhibition of Cas9‐mediated DNA cleavage was observed regardless of whether AcrIIA13b was added before or after the formation of the Cas9‐sgRNA RNP complex (Fig. 3B). These results indicate that the inhibitory effect of AcrIIA13b is independent of the order of component addition and suggest that AcrIIA13 can exert its function even after RNP assembly, likely through a mechanism that does not require disruption of complex formation.

Next, we examined whether AcrIIA13b could inhibit Cas9 even after the initiation of R‐loop formation. To test this, we pre‐incubated Cas9 and sgRNA to form a stable Cas9 RNP complex, then added AcrIIA13b either before or after introducing the target DNA. All reactions were initiated by adding Mg^2+^ after all components were assembled, allowing us to capture a pre‐cleavage state where R‐loop formation is likely initiated but not stabilized. Regardless of the order of addition, AcrIIA13b consistently inhibited Cas9‐mediated DNA cleavage (Fig. 3C). These results indicate that AcrIIA13b can interfere with Cas9 activity not only before DNA binding but also after the onset of R‐loop formation, although its action likely occurs before a fully stable R‐loop is established.

### AcrIIA13b binds directly to Cas9 via the WED and PI domains

**Fig. 4:**
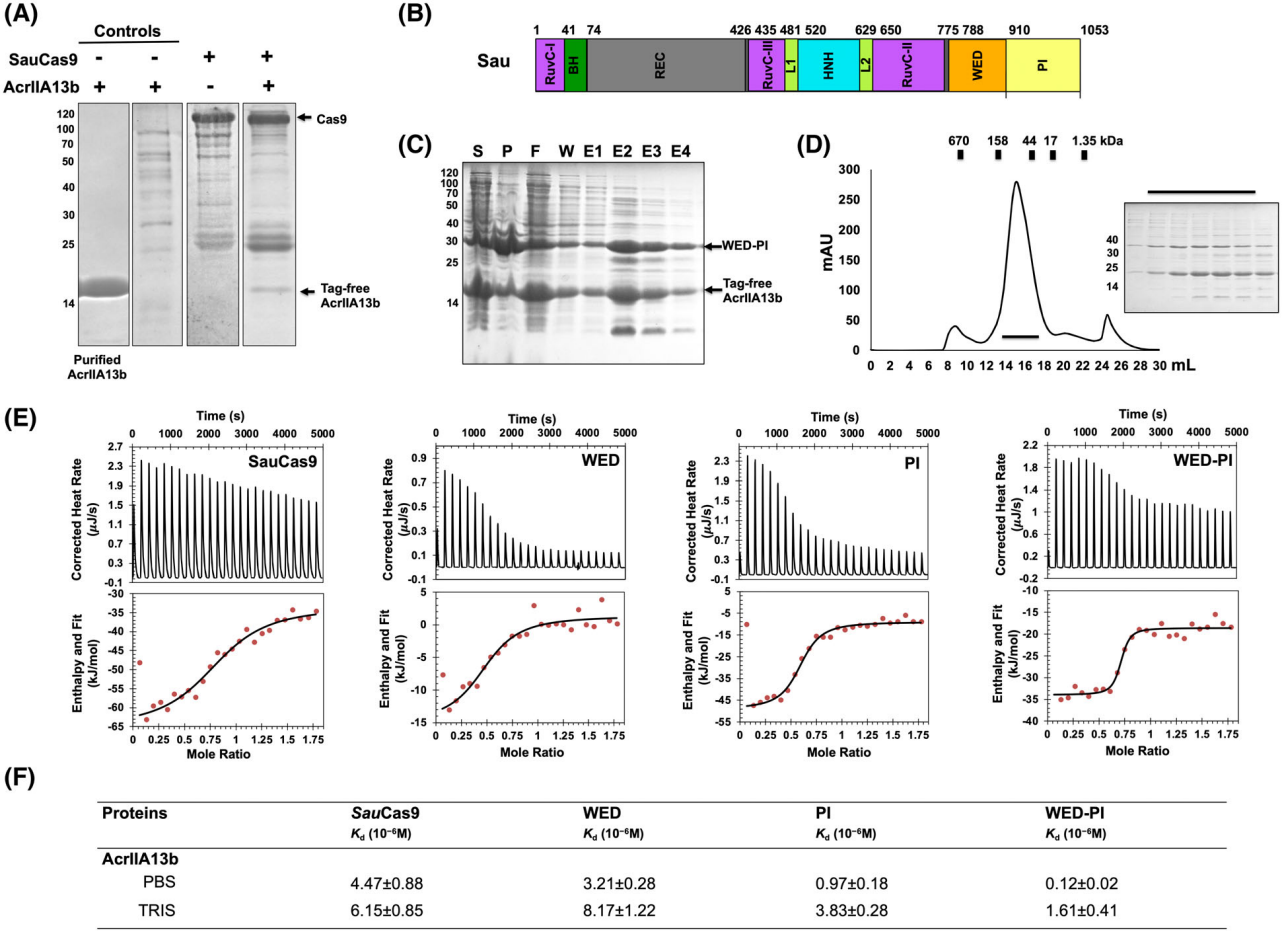
AcrIIA13b directly binds Cas9 via WED and PI domains. (A) Interaction analysis between tag‐free AcrIIA13b and His‐tagged SauCas9 by pull‐down assay, followed by sodium dodecyl sulfate/polyacrylamide gel electrophoresis (SDS/PAGE) analysis. SDS/PAGE gels produced by loading one of the main fractions from the Cas9 sample with (+AcrIIA13b) or without (−AcrIIA13b) providing AcrIIA13b, as well as a sample containing tag‐free AcrIIA13b alone, which served as a negative control. AcrIIA13b that pulled down by Cas9 is indicated by a black arrow. Representative of three experiments (*n* = 3). (B) Domain organization of SauCas9. (C) Affinity chromatography of His‐tagged WED‐PI co‐expressed tag‐free AcrIIA13b. E, elution; F, flow‐through; P, pellet after cell lysis; S, supernatant; W, washing. Representative of three experiments (*n* = 3). (D) Size‐exclusion chromatography (SEC) profile of pull‐downed elution fractions obtaining from affinity chromatography. An SDS/PAGE gel loaded with the peak fractions (fractions 10~16) from the SEC profile is provided to the right. Loaded fractions are indicated by the horizontal black bar. The corresponding fractions from SEC loaded onto SDS/PAGE gels are indicated by black bar. Representative of three experiments (*n* = 3). (E) Isothermal titration calorimetry (ITC) experiment showing titration of a *Sau*Cas9, WED, PI, and WED‐PI solution with AcrIIA13b in the PBS buffer condition. ITC was performed at 20 °C. The raw ITC data are shown in the upper panel, and experimental fitting of the data to a single‐site interaction model is shown in the lower panel. Representative of three experiments in each condition (*n* = 3). (F) Summary table of ITC experiment results of AcrIIA13b with SauCas9, WED, PI, and WED‐PI in Tris and PBS buffers.

Various strategies employed by Acr proteins to inhibit Cas9 include direct interactions with target DNA [40], sgRNA [41, 42, 43], or Cas9 itself [7, 24, 27, 32, 44, 45, 46, 47, 48]. To investigate the mechanism of AcrIIA13b inhibition, we conducted binding assays with DNA, RNA, and full‐length Cas9. AcrIIA13b did not induce any noticeable band shifts with nucleic acids, indicating a lack of direct interaction (Fig. S1A and B). In contrast, pull‐down assays confirmed direct binding to SauCas9, the known target of AcrIIA13. Specifically, tag‐free AcrIIA13b was efficiently pulled down by His‐tagged SauCas9 (Fig. 4A), supporting a protein–protein interaction as the primary inhibitory mechanism.

SauCas9 comprises several functional domains, including REC, HNH, WED, and PI (Fig. 4B). To determine which of the domains mediate the interaction with AcrIIA13b, we cloned and expressed each domain individually and assessed binding by pull‐down assays (Fig. S2). AcrIIA13b exhibited slight interaction with WED and PI domains individually but showed strong binding when both domains were present together, as seen in the WED‐PI construct. In contrast, no interaction was detected with the REC and HNH domains (Fig. S2). These findings suggest that the WED and PI domains cooperatively contribute to AcrIIA13b binding.

To further confirm this interaction, affinity chromatography was performed using co‐expression of the His‐tagged WED‐PI and tag‐free AcrIIA13b, showing efficient pull‐down of AcrIIA13b (Fig. 4C). Additionally, SEC experiments using samples eluted from affinity chromatography demonstrated the simultaneous co‐migration of AcrIIA13b with WED‐PI (Fig. 4D), providing sufficient evidence that AcrIIA13b indeed binds between the WED and PI domains of SauCas9.

To quantify these interactions, we conducted isothermal titration calorimetry (ITC) with full‐length SauCas9 and its individual domains (WED, PI, and WED‐PI) under both PBS and Tris buffer conditions. The two buffers were selected to assess protein stability and optimize assay sensitivity. Although the dissociation constants (*K*_d_) varied slightly between buffers, both conditions yielded consistent binding trends. PBS buffer generally resulted in lower *K*_d_ values, indicating stronger apparent binding and improved detection of binding heat. All interactions fit well to a single‐site binding model, showing no cooperative interaction (Fig. 4E). The calculated *K*_d_ values in PBS buffer were 4.47 μm for SauCas9, 3.21 μm for WED, 0.97 μm for PI, and 0.12 μm for WED‐PI in PBS buffer, confirming that AcrIIA13b exhibits the strongest affinity toward the WED‐PI domain (Fig. 4F).

Together, these binding results demonstrate that AcrIIA13b directly binds to SauCas9, primarily through the WED and PI domains to exert its inhibitory activity. This comprehensive analysis provides clear insight into the specific interactions involved in the inhibition mechanism of AcrIIA13b.

### Binding mode analysis indicated that the acidic surface formed by E31 and E39 is critical for the binding to Cas9

**Fig. 5:**
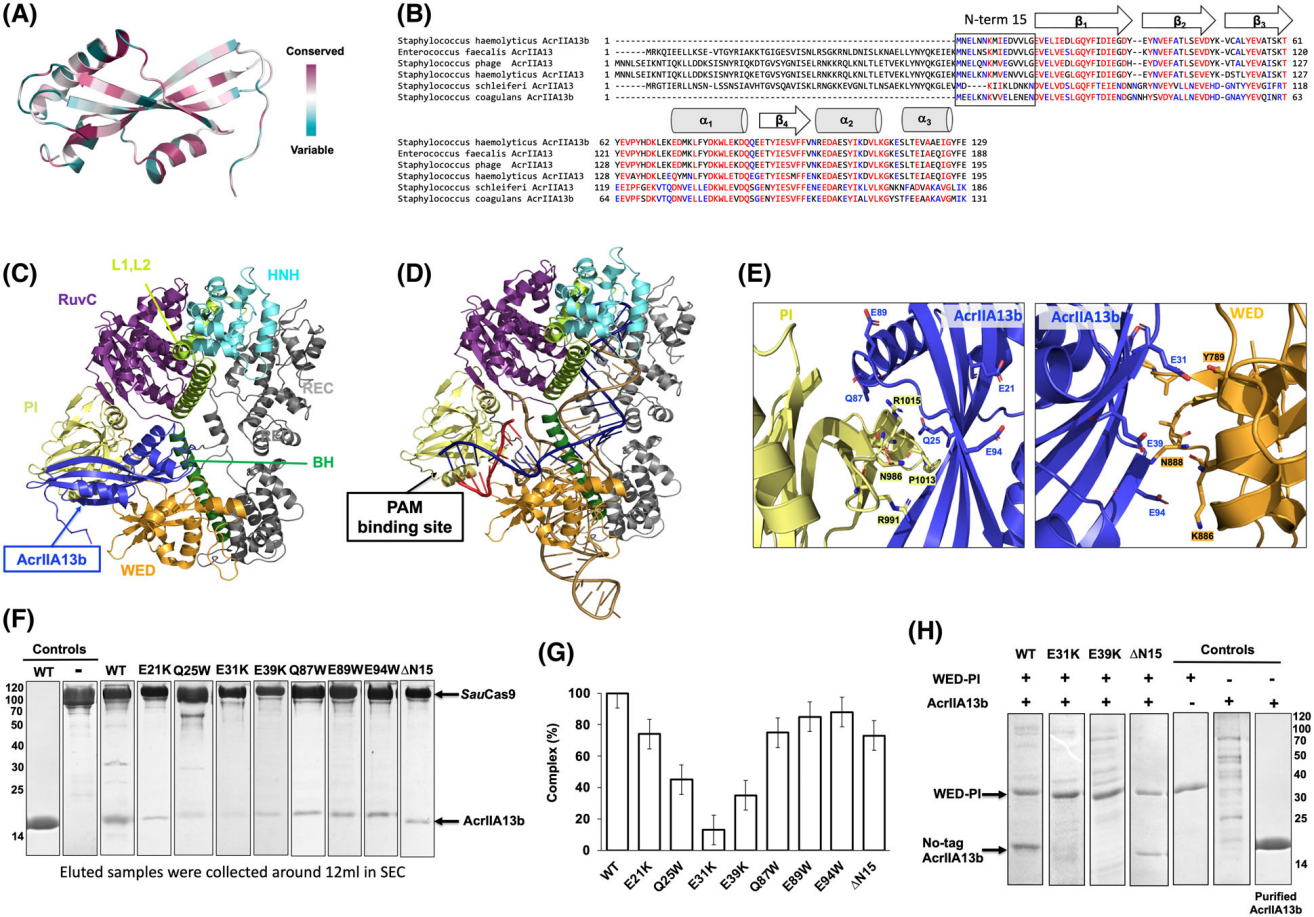
Acidic patch formed by E31 and E39 of AcrIIA13b is critical for the binding to Cas9. (A) Graphical representation of AcrIIA13b by pymol, colored relative to the amino acid sequence conservation degree generated by the ConSurf server. (B) Sequence alignment of AcrIIA13b and AcrIIA13 from different species. Accession numbers: *Staphylococcus haemolyticus* AcrIIA13b (WP_261007165), *Enterococcus faecalis* AcrIIA13 (WP_228082016), *Staphylococcus phage* AcrIIA13 (ARM678220.1), *Staphylococcus haemolyticus* AcrIIA13 (WP_142379361), *Staphylococcus coagulans* AcrIIA13b (WP_214892735), and *Staphylococcus schleiferi* AcrIIA13 (SUM89085.1). Alignment was generated using clustal omega. The N‐terminal 15 residues are indicated with a black box. (C) Docking cartoon model of AcrIIA13b docked onto Cas9 using HDOCK and visualized using pymol. (D) Cartoon model of Cas9 complexed with single‐guide RNA (sgRNA) and target DNA, visualized using pymol. The PAM‐recognition site located between WED and PI domains is indicated by a black arrow. (E) Analysis of protein–protein interactions (PPI) detail from the docking model of AcrIIA13b/Cas9 complex. Visualization in pymol. The left is magnified view of PPI between PI domain of Cas9 and AcrIIA13b and the right is magnified view of PPI between WED domain of Cas9 and AcrIIA13b. (F) Interaction analysis between Cas9 and various PPI‐disrupting mutants of AcrIIA13b by size‐exclusion chromatography (SEC), followed by sodium dodecyl sulfate/polyacrylamide gel electrophoresis (SDS/PAGE) analysis. SDS/PAGE gels produced by loading one of the main fractions (eluted around 12 mL) from the Cas9 sample with wild‐type or various mutants are shown. AcrIIIA13b mutants that co‐migrated with Cas9 indicated by a black arrow. Representative of three experiments (*n* = 3). The control purified AcrIIA13b gel shown here was used as a positional control and is the same gel as that presented in Fig. 4A. (G) Bar chart showing the quantified intensity of the co‐eluted AcrIIA13b mutants. Data are presented as the mean ± standard deviation of three independent experiments (*n* = 3). (H) Pull‐down analysis of two putative PPI‐disrupting tag‐free AcrIIA13b mutants, E31K, E39K and ∆N15, with WED‐PI. Representative of three independent experiments (*n* = 3). The control purified AcrIIA13b gel shown here was used as a positional control and is the same gel as that presented in Fig. 4A.

Next, we sought to determine how AcrIIA13b interacts with Cas9. To identify the critical residues responsible for AcrIIA13b's inhibitory activity and understand its mode of action, sequence conservation analysis was performed using the ConSurf server [49]. This analysis revealed that fully conserved residues were distributed throughout the AcrIIA13b structure (Fig. 5A,B).

To gain structural insight into how AcrIIA13b engages the WED and PI domains of Cas9, we generated a docking model of the AcrIIA31b‐SauCas9 complex (SauCas9 structure from PDB ID: 5CZZ [50]) using the HDOCK server [51]. The top‐ranked model, generated without grid restriction, positioned AcrIIA13b between the WED and PI domains of SauCas9, consistent with our *in vitro* interaction data (Fig. 5C).

To further support our docking model, we compared it with two independently derived structures. A model generated using alphafold2 showed a highly similar binding interface, positioning AcrIIA13b at the same WED‐PI interface as in the HODCK prediction (Fig. S3A and B). Moreover, structural alignment with the crystal structure of SauCas9 in complex with AcrIIA13 from *Staphylococcus schleiferi* (PDB ID: 7ENI) revealed that AcrIIA13 is also positioned between WED and PI domains (Fig. S4). These consistent results from independent modeling and structural data support the validity of our docking model and suggest a conserved binding mechanism. Notably, the predicted binding site overlaps with the region where the PAM‐containing target DNA is accommodated, implying that AcrIIA13b may inhibit Cas9 activity by sterically blocking PAM recognition (Fig. 5C,D).

Additionally, the dimer docking model based on the dimeric structure of AcrIIA13b was also successfully generated without steric clashes, supporting the structural feasibility of AcrIIA13b dimer binding (Fig. S5).

With this tentative AcrIIA13b/SauCas9 complex model, we analyzed the PPI. This analysis showed that Q25, Q87, and E89 of AcrIIA13b were involved in the interaction with the PI domain of SauCas9, while E31, E39, and E94 were involved in the interaction with the WED domain of SauCas9 (Fig. 5E). Based on the conserved residues identified in the sequence alignment and promising binding regions in the complex model, we confirmed the proposed model of the AcrIIA13b‐SauCas9 complex by selecting specific residues for mutagenesis and analyzing their effect on complexation. The chosen residues were mutated to opposite charges or bulky residues to potentially disrupt interactions with SauCas9. The E21K mutation was included as a negative control, while E31K, E39K, and E94W targeted the WED domain, and Q25W, Q87W, and E89W targeted the PI domain.

The results of the SauCas9 binding test revealed that the E31K and E39K mutants exhibited significantly reduced affinities for SauCas9 compared to the wild‐type AcrIIA13b (Fig. 5F,G and Fig. S6A‐I). The bar graph showing the quantitative analysis of the co‐migration supported this observation, confirming diminished binding for E31K and E39K (Fig. 5H).

The disruption was further confirmed by pull‐down analysis and ITC. Specifically, pull‐down assays using the WED‐PI domain showed that E31K and E39K mutants had markedly reduced interaction compared to wild‐type (Fig. 5H). In ITC experiments, both mutants showed significantly decreased binding affinity toward full‐length SauCas9, with thermograms that could not be fitted to a single‐site model. For WED‐PI, E31K showed no detectable binding, while E39K displayed a *K*_d_ of 1.31 μm, more than tenfold weaker than that of wild‐type (Fig. S7A‐D). These results confirm that E31 and E39 are key residues for WED‐PI domain interaction and the inhibitory function of AcrIIA13b.

To assess the role of AcrIIA13b dimerization in direct binding to Cas9, we analyzed the ∆N15 mutant, which lacks the N‐terminal 15 residues required for dimer formation. Binding assays, including SEC co‐migration with SauCas9 (Fig. 5F,G and Fig. S6A‐I) and pull down with the WED‐PI domain (Fig. 5H), showed that ∆N15 retained detectable, though slightly reduced, binding compared to wild‐type. To further determine whether the N‐terminus directly participates in the interaction, we performed structural modeling using HDOCK, which positioned the N‐terminal region distal to the WED‐PI domain (Fig. S8A‐C). Superposition with the 7ENI structure similarly showed that the N‐terminal residues do not extend toward SauCas9 (Fig. S4A and B). Together, these results suggest that the N‐terminal region is not structurally essential for Cas9 binding.

### Direct interaction with Cas9 and N‐terminal region contribute to AcrIIA13b inhibition

**Fig. 6:**
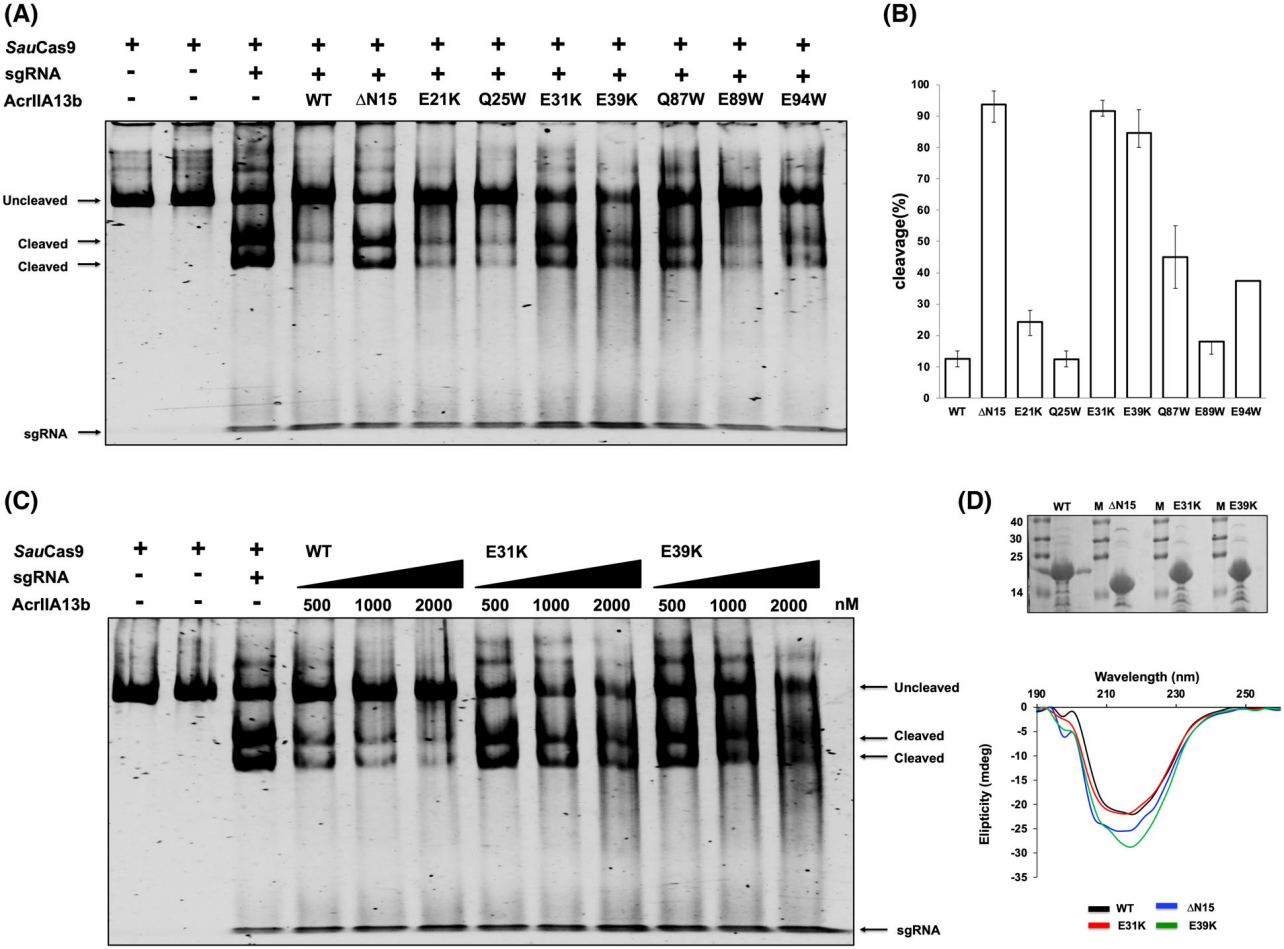
Direct interaction and dimerization of AcrIIA13b is critical for Cas9 inhibition. (A) *In vitro* target DNA cleavage assay using wild‐type Cas9 and various AcrIIA13b mutants. ‘+’ and ‘–’ indicate added and not added to the enzyme reaction, respectively. Representative of three experiments (*n* = 3). (B) Quantitative histogram of target DNA cleavage ratio according to (A). Data are presented as mean ± standard deviation from three independent *in vitro* target DNA cleavage assays (*n* = 3). (C) *In vitro* target DNA cleavage inhibition assay with different concentrations of AcrIIA13b and its mutants. ‘+’ and ‘–’ indicate added and not added to the enzyme reaction, respectively. Representative of three experiments (*n* = 3). (D) The experimental results of circular dichroism. The final purified protein samples that are used for circular dichroism (CD) experiments are shown. Representative of one experiment (*n* = 1), performed as a preliminary biophysical characterization to confirm proper protein folding.

The proposed inhibition mechanism of AcrIIA13b via direct interaction with the WED and PI domains of Cas9 was validated through a DNA cleavage assay using AcrIIA13b mutants. This assay was performed under consistent conditions, with the only varying being the AcrIIA13b variant used. Compared to the wild‐type AcrIIA13b, which strongly inhibited Cas9‐mediated DNA cleavage, the ΔN15, E31K, and E39K mutants displayed stronger cleaved bands, indicating a weakened inhibition of Cas9 (Fig. 6A,B). As E31 and E39 were identified as key residues, their impaired inhibition was further confirmed by concentration‐dependent analysis. Even at high concentration (2 μm), the E31K and E39K mutants failed to inhibit Cas9, whereas wild‐type AcrIIA13b strongly blocked its activity (Fig. 6C), confirming the critical role of direct binding in AcrIIA13b function.

To assess whether the reduced activity was due to altered protein folding, CD spectroscopy was performed. The results showed that all three mutants had CD spectra nearly identical to the wild‐type (Fig. 6D), indicating that their loss of activity was not caused by misfolding, but by disruption of functional interactions.

Interestingly, the ΔN15 mutation, which was designed to disrupt dimerization, also resulted in the complete loss of inhibitory activity (Fig. 6A,B). However, our previous SEC and pull‐down assays demonstrated that ΔN15 still retained binding to both full‐length SauCas9 and the WED‐PI domain (Fig. 5F–H), suggesting that the N‐terminal 15 residues are required for direct binding. To explore this discrepancy, we compared predicted complex structures of wild‐type and ΔN15 AcrIIA13b bound to the WED‐PI domain. These *in silico* models revealed no direct contacts between the N‐terminal residues and the binding interface, but truncation caused a subtle shift in the orientation of the WED domain (Fig. S9A‐C). These observations suggest that while the N‐terminal region does not directly participate in binding, it may influence the overall configuration of the inhibitory complex. Furthermore, the impaired activity of ΔN15 supports the possibility that AcrIIA13b dimerization, which is abolished in this mutant, also plays a functional role.

Together, these findings indicate that effective Cas9 inhibition by AcrIIA13b requires more than simple binding. While direct interaction, particularly through residues such as E31 and E39, is essential, additional structural features including the N‐terminal region and dimeric architecture also appear to contribute to the formation of a fully inhibitory complex.

### Proposed model of Cas9 inhibition by AcrIIA13b: mimicry of PAM duplex

The PAM‐recognition mechanism of *Sau*Cas9 is well characterized. Residues in the PI domain (N991, N986, N985, and R1015) interact with the major groove of the PAM duplex, while residues in the WED domain (Y789, Y882, K886, N888, and L909) engage the phosphate backbone of the PAM duplex [50]. Structural modeling of the AcrIIA13b‐SauCas9 complex revealed that AcrIIA13b residues E31, E39, and E94 are positioned near the PAM‐recognizing residues in the WED domain, while Q25 and Q87 are located adjacent to key residues in the PI domain (Fig. 5E). These spatial proximities suggest that AcrIIA13b may inhibit Cas9 activity by mimicking the PAM duplex and competitively occupying its binding pocket between the WED and PI domains, thereby preventing target DNA recognition.

**Fig. 7:**
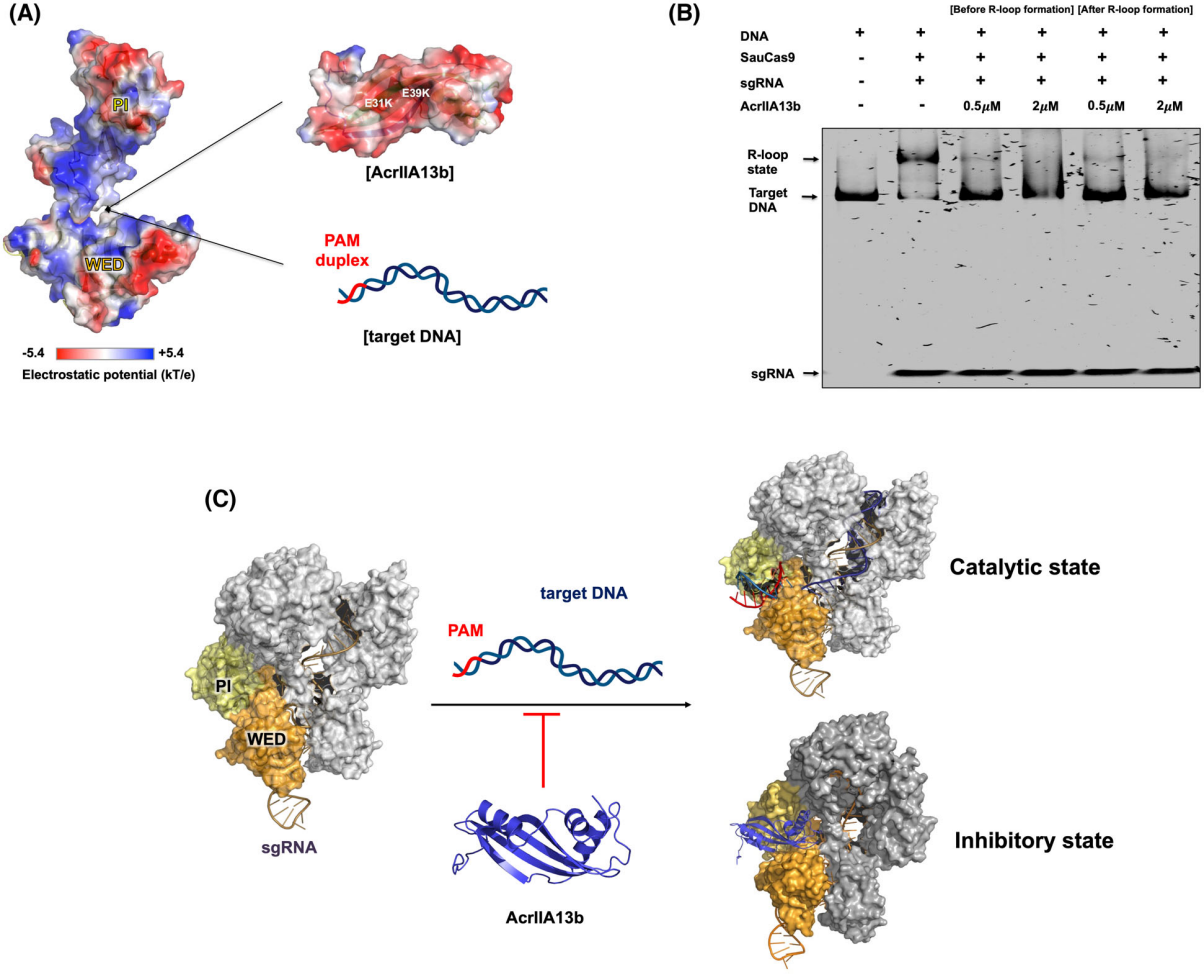
Proposed model of Cas9 inhibition by AcrIIA13b. (A) Features of surface charge of WED‐PI domain in SauCas9 (left) and AcrIIA13b (right), visualized in pymol. (B) Electrophoretic mobility shift assay (EMSA) analysis of the Cas9‐sgRNA‐DNA ribonucleoprotein (RNP) complex. ‘+’ and ‘–’ indicate added and not added to the enzyme reaction. The gel shows shifts corresponding to Cas9‐DNA complex formation, with decreased band intensity indicating AcrIIA13b‐mediated inhibition. Representative of three experiments (*n* = 3). (C) Proposed model of Cas9 inhibition by AcrIIA13b. AcrIIA13b (blue) is positioned at the WED‐PI interface of Cas9 (orange/yellow), in proximity to the PAM duplex region on target DNA (red/sky blue). Each component of the model was generated in pymol and assembled to illustrate the proposed inhibitory mechanism.

Electrostatic surface analysis supports this mechanism. The PAM‐interacting pocket between the WED and PI domains displays a predominantly positive potential, while the corresponding surface of AcrIIA13b is negatively charged (Fig. 7A). This electrostatic complementarity resembles the native interaction between Cas9 and the PAM duplex, further supporting a mimicry‐based inhibitory mechanism.

To validate this model, EMSA was conducted to assess the impact of AcrIIA13b on target DNA binding to the Cas9 RNP complex. Incubation of SauCas9‐sgRNA with target DNA resulted in a shifted band corresponding to the RNP‐DNA complex in the pre‐cleavage R‐loop state (Fig. 7B). However, the formation of this complex was markedly reduced when AcrIIA13b was present, regardless of whether it was added before or after R‐loop formation. This suggests that AcrIIA13b not only blocks the initial loading of target DNA but may also displace pre‐bound DNA from the RNP complex (Fig. 7B), reinforcing its role in disrupting DNA engagement.

Taken together, our structural and biochemical data converge on a model in which AcrIIA13b inhibits SauCas9 by mimicking the PAM duplex and occupying the positively charged PAM‐binding pocket. Through both steric hindrance and electrostatic interactions, AcrIIA13b effectively prevents or reverses target DNA binding, thereby abolishing Cas9 cleavage activity (Fig. 7C). This mechanism offers new insight into CRISPR‐Cas regulation and expands our understanding of anti‐CRISPR protein function.

## Discussion

AcrIIA13 was initially identified from *Staphylococcus schleiferi* as a potent type II‐A Acr protein that inhibits SauCas9 and acts as a transcriptional regulator via a conserved HTH domain. Its homolog, AcrIIA13b from *St. haemolyticus*, lacks this HTH motif but still exhibits strong inhibitory activity against SauCas9 [33]. In this study, we determined the crystal structure of AcrIIA13b and found that it adopts a unique fold with four β‐sheets and three α‐helices. Despite being monomeric in the crystal form, solution‐based analyses and PISA calculations indicated that it forms a symmetric dimer. Mutational disruption of the N‐terminal beta‐strand (ΔN15) confirmed the importance of this region for dimerization. The variable SEC profiles observed during purification may reflect a dynamic equilibrium between monomeric and dimeric forms.

Mechanistically, AcrIIA13b functions by occupying the PAM‐binding interface, particularly engaging the WED and PI domains. This mode of inhibition resembles that of other type II Acrs that block target DNA loading by interfering with PAM recognition [25, 44, 45, 52]. The docking models of the AcrIIA13b‐Cas9 complex aligned closely with the experimentally solved structure of the AcrIIA13‐SauCas9 complex (PDB ID: 7ENI), supporting the structural accuracy of our predictions and underscoring the conserved nature of this inhibitory interface. Supporting this, EMSA experiments showed that AcrIIA13b inhibits the formation of the Cas9 RNP‐DNA complex and can even displace target DNA after pre‐cleavage R‐loop formation, indicating both preventive and disruptive modes of inhibition.

The ΔN15 mutant, while still capable of Cas9 binding, failed to inhibit its activity, highlighting the functional importance of the N‐terminal region. Structural modeling revealed altered binding orientation in this mutant, particularly with the WED‐PI domain, suggesting a role in stabilizing the inhibitory interface or maintaining the proper geometry for effective binding. This is reminiscent of other Acrs where dimerization contributes to enhanced inhibitory function, as in AcrIIA6 or AcrIIC3 [32, 38]. However, in our study, the direct mechanistic link between dimerization and inhibitory activity remains to be fully validated and is instead suggested based on structural observations and loss‐of‐function mutation data.

Given the increasing use of SauCas9 due to its compact size and multi‐turnover capability [53, 54], understanding its specific inhibitors is crucial. Our study establishes AcrIIA13b as a potent inhibitor that functions through a PAM mimicry mechanism. This adds to the repertoire of inhibitory strategies employed by Acrs and provides a framework for engineering precision regulators of Cas9 activity.

In conclusion, we provide a comprehensive structural and mechanistic characterization of AcrIIA13b. Its inhibitory action depends not only on direct interaction with Cas9 but also on structural features such as dimerization and an intact N‐terminal region. These findings deepen our understanding of anti‐CRISPR regulation and offer valuable insights for the design of next‐generation CRISPR control systems.

## Materials and methods

### Cloning, overexpression, and purification of AcrIIA13b, various Cas9, and various Cas9 domains for structural and biochemical studies

The overall experimental procedures used in cloning and protein purification followed well‐established methods previously developed and routinely applied in our laboratory [7, 24]. Primer sequences used in this study are listed in Table S2. The full‐length *acrIIA13b* (residues 1–129) gene from *St. haemolyticus* (accession number: WP_053038109.1) was synthesized by Bionics (Daejeon, Korea), then cloned into a pET21a plasmid vector (Novagen, Madison, WI, USA), which contains a C‐terminal hexa (6×)‐histidine tag for affinity chromatography. The *Nde*I and *Xho*I restriction sites were used for cloning. After transforming the resulting recombinant construct into *Escherichia coli* BL21 (DE3) competent cells, a single colony was selected and cultured at 37 °C in 1 L of lysogeny broth containing 50 μg·mL^−1^ of ampicillin until the optical density at 600 nm reached 0.7–0.8. Then, 0.25 mm isopropyl‐β‐d‐1‐thiogalactopyranoside (IPTG) was added and incubated for 18 h in a shaking incubator at 20 °C. The cultured cells were harvested by centrifugation at 2350 ***g*** for 15 min at 20 °C, resuspended in 20 mL of lysis buffer (20 mm Tris/HCl, pH 8.0; 500 mm NaCl) and lysed under ultrasonication at 4 °C. The cell lysate and the supernatant were separated by centrifugation at 27 000 ***g*** at 4 °C for 30 min. The supernatants were pooled, mixed with nickel‐nitrilotriacetic acid (NTA) affinity resin, and incubated at 4 °C for 2 h. Then, the incubated mixture was loaded onto a gravity‐flow column (Bio‐Rad, Hercules, CA, USA). After the mixture was passed through the resin, the resin was washed with an additional 50 mL of lysis buffer to remove the impurities, and the resin‐bound AcrIIA13b was then eluted with elution buffer (20 mm Tris/HCl, pH 8.0; 500 mm NaCl, 250 mm imidazole). The AcrIIA13b was further purified by size‐exclusion chromatography (SEC) on a Superdex 200 Increase 10/300 GL column (GE Healthcare, Waukesha, WI, USA) previously equilibrated with SEC buffer (20 mm Tris/HCl pH 8.0; 150 mm NaCl) and connected to an ÄKTA Explorer system (GE Healthcare). Eluted fractions were collected, pooled, and finally concentrated to 7.6 mg·mL^−1^ for crystallization. The purity of the sample was visualized using sodium dodecyl sulfate/polyacrylamide gel electrophoresis (SDS/PAGE).

The expression constructs of *Staphylococcus aureus* Cas9 (#101086) were purchased from Addgene. Expression constructs for the various recognition (REC) domains, the HNH domain, the wedge‐like (WED) domain, and the PAM‐interacting (PI) domain were produced by cloning using the purchased Cas9 plasmid as a template. All the PCR products were cloned into the pET21a vector using *Nde*I and *Xho*I restriction sites. All the proteins were purified using the same method as that used for AcrIIA13b purification.

### Crystallization and X‐ray diffraction data collection

The overall experimental procedures used in crystallization followed well‐established methods routinely applied in our laboratory [24]. Briefly, the crystallization of AcrIIA13b was carried out using the hanging drop vapor diffusion method and incubated at 20 °C. Initial crystals were obtained by mixing 1 μL of protein solution (7.0 mg·mL^−1^ protein in SEC buffer) with 1 μL of reservoir solution (1.6 m sodium citrate) and equilibrating the mixture against 0.3 mL of reservoir solution. To optimize the crystallization conditions, a further optimization step was performed using 1.2 m sodium citrate pH 6.1, resulting in the formation of the best crystal within 3 days. Subsequently, a single crystal was selected and soaked in the reservoir solution supplemented with 30% (v/v) glycerol for cryoprotection. X‐ray diffraction data were collected at −178 °C on the BL‐5C beamline at the Pohang Accelerator Laboratory (Pohang, Korea). Data processing, which included indexing, integration, and scaling, was performed using hkl2000 software [55].

### Structure determination and refinement

The structure of AcrIIA13b was determined using molecular replacement (MR) phasing, employing the phaser program within the phenix package [56]. The initial search model for MR was generated using the predicted structural model from alphafold2 [34, 57]. The initial model was automatically built using the autobuild module in phenix. Subsequent refinement and model building were performed using coot [58] and phenix.refine [56]. The quality and stereochemistry of the final structure were validated using molprobity [59]. All structural figures were created using pymol [60].

### Multi‐angle light scattering (MALS) analysis

The overall experimental procedures used in MALS analysis followed well‐established methods routinely used in our laboratory [7, 24]. Briefly, purified AcrIIA13b protein solution was loaded onto a 24 mL Superdex 200 Increase 10/300 GL column that had been pre‐equilibrated with SEC buffer. SEC‐MALS was performed at 20 °C with a buffer flow rate of 0.5 mL·min^−1^. The ÄKTA Explorer system (GE Healthcare) was coupled with a DAWN TREOS MALS detector (Wyatt Technology, Santa Barbara, CA, USA). For calibration, a reference molecular mass value was established using bovine serum albumin. Data were processed and assessed using astra software (Wyatt Technology).

### Mutagenesis

The overall experimental procedures used in mutagenesis followed well‐established methods routinely used in our laboratory [7]. Site‐directed mutagenesis was conducted using a QuikChange kit (Stratagene, San Diego, CA, USA) according to the manufacturer's instructions. The resulting mutations were validated by sequencing to ensure the accuracy of the introduced changes. Primer sequences used for mutagenesis are listed in Table S2. All mutant proteins were subsequently expressed and purified using the same methodology described earlier for the wild‐type protein.

### Sequence alignment

The amino acid sequences of AcrIIA13 homologs across different species were analyzed using clustal omega [61].

### Size‐exclusion chromatography (SEC) assay for complex formation

The overall experimental procedures used in the SEC assay followed well‐established methods previously developed and routinely used in our laboratory [24]. To investigate the formation of complexes between Cas9/AcrIIA13b, SEC was employed. AcrIIA13b variants were mixed with Cas9 protein, followed by incubation for 1 h at 4 °C. The resulting mixture was then loaded onto a Superdex 200 Increase 10/300 GL column (GE Healthcare) that had been pre‐equilibrated with SEC buffer (20 mm Tris/HCl, pH 8.0; 150 mm NaCl). The eluted fractions corresponding to the peaks of interest were collected, and the samples were subsequently analyzed by SDS/PAGE, followed by staining the SDS/PAGE gel with Coomassie Brilliant Blue. The migration patterns and co‐migration of protein bands within the gel were carefully examined and analyzed to assess the complex formation between AcrIIA13b mutant variants and Cas9. To quantify complex formation, ‘Complex (%)’ was calculated by measuring the band width (intensity) of the co‐migrated AcrIIA13b band relative to the corresponding Cas9 band in each lane. All values were normalized to the wild‐type control to enable comparison across samples while minimizing variability.

### *In vitro* anti‐CRISPR activity assay

To evaluate the inhibitory activity of AcrIIA13b and its mutants on Cas9‐mediated target cleavage, *in vitro* target DNA cleavage assays were performed. *Sau*Cas9 (30 nm), purchased from NEB (#M0654T), was mixed with a sgRNA (33 nm) synthesized using the HiScribe™ T7 Quick High Yield RNA Synthesis Kit (NEB) and incubated in NEBuffer r3.1 (NEB) for 20 min at 25 °C. Subsequently, 0.5 to ~ 2 μm of wild‐type AcrIIA13b and 2 μm of its mutants (ΔN15, E21K, Q25W, E31K, E39K, Q87W, E89W, and E94W) were added to the Cas9/sgRNA mixture and incubated for an additional 20 min at 25 °C. Following this incubation step, target DNA (1.4 nm) was introduced into the reaction mixture, and the reaction was carried out at 37 °C for 30 min. To halt the reaction, proteinase *K* was added and incubated for 10 min. All reaction products were then loaded on 4% polyacrylamide gels and separated by electrophoresis. Visualization of the DNA cleavage products was achieved by staining the gels with SYBR Gold. The resulting gel images were analyzed to assess the inhibitory effect of wild‐type AcrIIA13b and its mutants on Cas9‐mediated target cleavage. Oligonucleotide sequences used in this study are listed in Table S2.

### Inhibition activity analysis of AcrIIA13b with SauCas9‐RNP vs. SauCas9‐RNP + DNA

SauCas9 and sgRNA were pre‐incubated at 25 °C for 20 min in a buffer containing 100 mm NaCl, 50 mm Tris/HCl pH 8.0, 100 μg·mL^−1^ BSA. To test on the ‘before R‐loop formation condition’, AcrIIA13b (0.5, 1, 2 μm) was added to SauCas9‐RNP, followed by the addition of 1.4 nm target DNA to SauCas9‐RNP‐AcrIIA13b. Each sample was then incubated at 25 °C for 20 min for each step. To test on the ‘after R‐loop formation condition’, 1.4 nm target DNA was added to form the SauCas9 RNP‐DNA complex first, and then the AcrIIA13b (0.5, 1, 2 μm) were added and incubated at 25 °C for 20 min for each step. Subsequently, 10 mm MgCl_2_ was added to all samples to initiate cleavage activity at 37 °C for 30 min. The reactions were analyzed using electrophoresis with 4% polyacrylamide gels.

### Isothermal titration calorimetry (ITC)

NanoITC (TA Instruments, New Castle, DE, USA) was utilized for ITC experiments. Protein samples for ITC were prepared in both PBS pH 7.4 and Tris/HCl 8.0 buffers. Before titration, all protein samples were centrifuged at 15 000 *g* for 20 min at 4 °C to remove any debris, then degassed for 20 min at 20 °C. For titration, concentrated AcrIIA13b (500 μm) was injected into a cell containing each target proteins, including SauCas9_WED‐PI domain (50 μm), SauCas9_WED, and SauCas9_PI *et al*. All titrations were performed at 20 °C with 25 injections at 200‐s intervals. Baseline controls were obtained by titrating AcrIIA13b into only buffer condition.

For the affinity measurement between AcrIIA13b (500 μm) and the full‐length Cas9s (50 μm), the protein samples were prepared in 20 mm Tris/HCl pH 8.0, 150 mm buffer, and for the baseline controls, AcrIIA13b was titrated into the buffer. Other conditions remained identical to the previous setup. The binding isotherms were analyzed using the software provided by TA Instruments.

### His‐tag pull‐down assay

Tag‐free AcrIIA13b was co‐transformed with His‐tagged full‐length Cas9 and each domain of Cas9 into *E. coli* strain BL21(DE3). The co‐transformed tag‐free AcrIIA13b and His‐tagged Cas9s, along with each domain, were cultured at 37 °C in lysogeny broth containing 50 μg·mL^−1^ of ampicillin and 50 μg·mL^−1^ of kanamycin until the A_600nm_ reached 0.7. The subsequent steps were performed similarly to those for the wild‐type AcrIIA13b, except for the use of 20 mm Tris/HCl pH 8.0 and 150 mm NaCl buffer for both resuspension buffer and the wash buffer during Ni‐NTA affinity chromatography. The pull‐downed tag‐free AcrIIA13b with His‐tagged full‐length Cas9 and His‐tagged domains (REC, HNH, WED, PI, and WED‐PI) was visualized using SDS/PAGE. As a negative control, tag‐free AcrIIA13b alone was also subjected to SDS/PAGE analysis to confirm the absence of non‐specific binding to the Ni‐NTA resin.

Subsequently, tag‐free AcrIIA13b and his‐tagged SauCas9_WED‐PI domain complex were further purified using size‐exclusion chromatography (SEC) on a Superdex 200 Increase 10/300 GL column (GE Healthcare, Waukesha, WI, USA), with SEC buffer (20 mm Tris/HCl pH 8.0; 150 mm NaCl), connected to an ÄKTA Explorer system (GE Healthcare). The co‐migrated tag‐free AcrIIA13b with SauCas9_WED‐PI was visualized using SDS/PAGE.

### Modeling of AcrIIA13b/ *Sau*Cas9 complex structure

To generate the AcrIIA13b/*Sau*Cas9 complex structure, the HDOCK server was employed [51]. The *Sau*Cas9 structure used for the docking process was obtained from a previously solved structure available from the Protein Data Bank (PDB ID 5CZZ) [50]. The default parameters provided by the HDOCK server were employed for the docking simulation. Additionally, complex models of SauCas9 with wild‐type and ΔN15 AcrIIA13b, as well as the WED‐PI domain, were predicted using alphafold3 with default settings [62].

### Circular dichroism spectroscopy

The wild‐type and mutated AcrIIA13b in PBS buffer were prepared at a concentration of 0.4–0.5 mg·mL^−1^. Each sample (200 μL) was scanned using a JASCO J‐1500 spectropolarimeter at the Korea Basic Science Institute (Ochang, South Korea) for a CD experiment. Spectra were obtained from 190 to 260 nm at 25 °C in a 1‐mm pathlength quart cuvette using a bandwidth of 1.0 nm, a scanning speed of 100 nm·min^−1^, a response time of 1 s, and three scans were accumulated and averaged.

### Target DNA EMSA with AcrIIA13b

SauCas9 (99 nm) and sgRNA (90 nm) were pre‐incubated with a buffer (100 mm NaCl, 50 mm Tris/HCl pH 8.0, and 100 μg·mL^−1^ BSA) at 37 °C for 10 min. To assess AcrIIA13b's ability to hinder complex formation depending on the presence or absence of the Cas9 RNP‐target DNA complex, AcrIIA13b (~ 4 μm) was added and incubated at room temperature for 20 min, followed by the addition of target DNA (1.4 nm), and further incubated at 37 °C for 10 min. After appropriately incubating each sample, they were loaded onto a 0.5× 4% TBE gel, and the target DNA was visualized using SYBR GOLD dye and documented using the FastGene FAS‐BG LED BOX.

## Conflict of interest

The authors declare no conflict of interest.

## Author contributions

HHP designed and supervised the project. SYL performed all the experiments, including collecting the biochemical and structural data and solving the structure. HHP and SYL wrote the manuscript. Both authors discussed the results and commented on the manuscript.

## Supporting information


**Fig. S1.** Binding analysis of AcrIIA13b to nucleic acids.
**Fig. S2.** Pull‐down assay of AcrIIA13b with SauCas9 domains.
**Fig. S3.** Comparison of docking model of SauCas9 and AcrIIA13b complex generated by HDOCK with that generated by alphafold2.
**Fig. S4.** Comparison of docking model of SauCas9‐AcrIIA13b complex and crystal structure of SauCas9‐AcrIIA13 complex (unpublished, PDBID: 7ENI).
**Fig. S5.** Modeled Cas9 dimerization induced by AcrIIA13b dimer.
**Fig. S6.** Analysis of the interactions between Cas9 and various mutants of AcrIIA13b on SEC followed by SDS/PAGE.
**Fig. S7.** ITC analysis of AcrIIA13b mutants E31K and E39K binding to SauCas9 and WED‐PI domain.
**Fig. S8.** Structural docking models of AcrIIA13b WT and ΔN15 mutant with SauCas9 generated by HDOCK server.
**Fig. S9.** alphafold3‐predicted complex models of AcrIIA13b WT and ΔN15 mutant with the WED‐PI domain of SauCas9.
**Table S1.** Table summarizing the result of structural similarity search using the Dali server.
**Table S2.** Oligonucleotides used in this study.

## Data Availability

The coordinate and structure factor have been deposited into the Research Collaboratory for Structural Bioinformatics (RCSB) Protein Data Bank (PDB) under the PDB code of 8K4M.
