# Supplementary material for: Investigating the molecular mechanisms underlying the anti‐CRISPR function of AcrIIA13b protein
Source: FEBS J. 2025 Oct 24;293(9):2560–78. doi: 10.1111/febs.70304 (PMC13147308; doi:10.1111/febs.70304)
Supplement: Supplementary file 1 — Fig. S1. Binding analysis of AcrIIA13b to nucleic acids. Fig. S2. Pull‐down assay of AcrIIA13b with SauCas9 domains. Fig. S3. Comparison of docking model of SauCas9 and AcrIIA13b complex generated by HDOCK with that generated by alphafold2. Fig. S4. Comparison of docking model of SauCas9‐AcrIIA13b complex and crystal structure of SauCas9‐AcrIIA13 complex (unpublished, PDBID: 7ENI). Fig. S5. Modeled Cas9 dimerization induced by AcrIIA13b dimer. Fig. S6. Analysis of the interactions between Cas9 and various mutants of AcrIIA13b on SEC followed by SDS/PAGE. Fig. S7. ITC analysis of AcrIIA13b mutants E31K and E39K binding to SauCas9 and WED‐PI domain. Fig. S8. Structural docking models of AcrIIA13b WT and ΔN15 mutant with SauCas9 generated by HDOCK server. Fig. S9. alphafold3‐predicted complex models of AcrIIA13b WT and ΔN15 mutant with the WED‐PI domain of SauCas9. Table S1. Table summarizing the result of structural similarity search using the Dali server. Table S2. Oligonucleotides used in this study. [file FEBS-293-2560-s001.pdf]

Investigating the molecular mechanisms underlying the anti-CRISPR function of AcrIIA13b protein

So Yeon Lee[1,2,] and Hyun Ho Park[1,2,*]

[1] College of Pharmacy, Chung-Ang University, Seoul 06974, Republic of Korea
[2] Department of Global Innovative Drugs, Graduate School of Chung-Ang University, Seoul 06974, Republic of Korea

* To whom correspondence should be addressed. Hyun Ho Park; College of Pharmacy, Chung-Ang University, Seoul 06974, Republic of Korea; Tel: +82-2-820-5930; Fax: +82-2-820-3033; Email: xrayleox@cau.ac.kr

Keywords
Anti-CRISPR; AcrIIA13b; Adaptive immunity; CRISPR-Cas system; Crystal structure

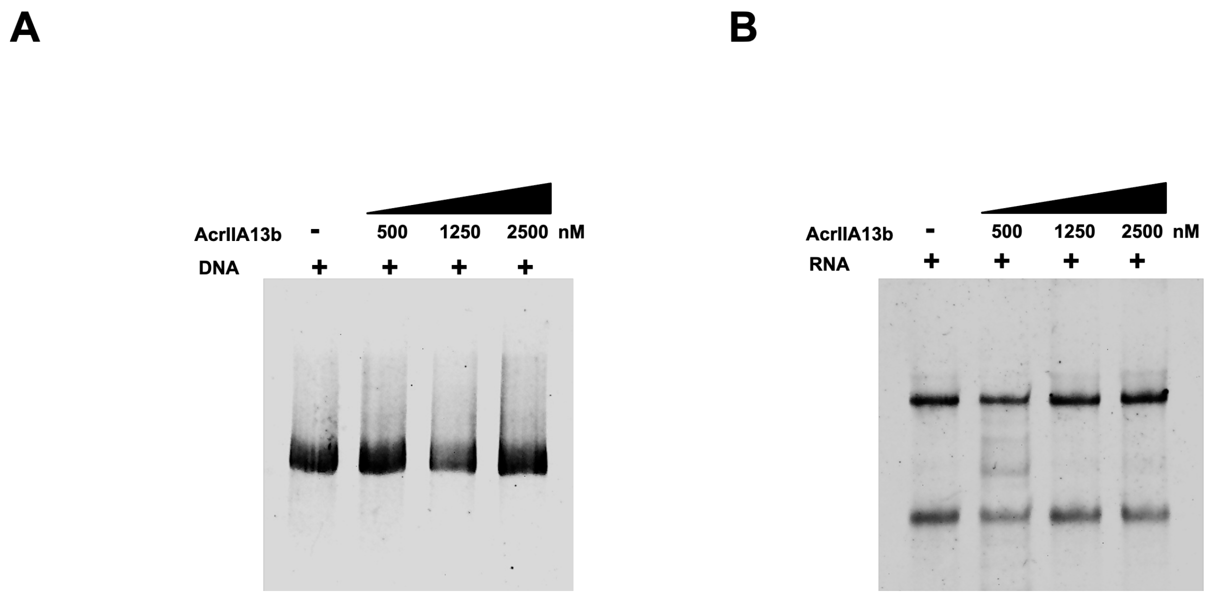

Supplementary Figure 1. Binding analysis of AcrIIA13b to nucleic acids. Validation of the interaction of AcrIIA13b to target DNA (A) and single-guide RNA (sgRNA) (B) using electrophoretic mobility shift assay (EMSA). Mobility shifts of the DNA and RNA were observed on the 4 % polyacrylamide gels stained by SYBR Gold. Representative of two experiments (n=2).

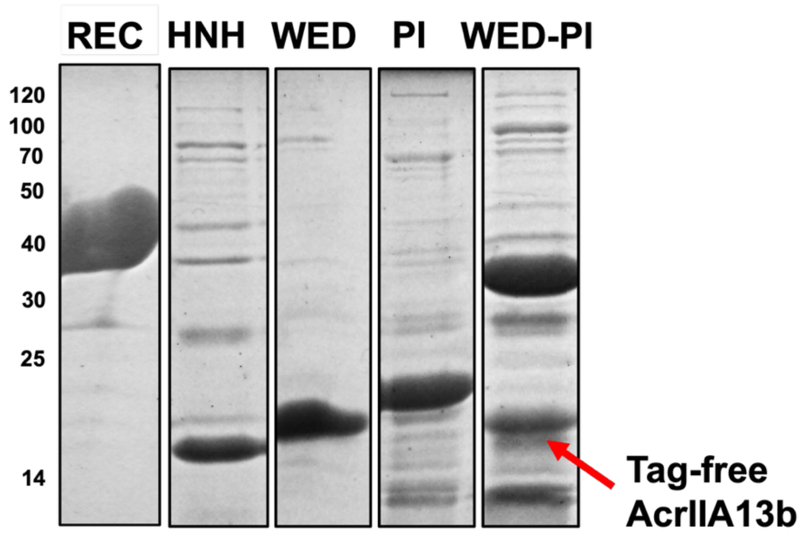

Supplementary Figure 2. Pull-down assay of AcrIIA13b with SauCas9 domains.
Pull-down analysis of various *Sau*Cas9 domains with tag-free AcrIIA13b. The distinctly pulled-down tag-free AcrIIA13b is indicated by the red arrow.
(Molecular weights: SauCas9_REC$_{6XHis}$,42.042kD; SauCas9_HNH$_{6XHis}$, 13.873kD; SauCas9_WED$_{6XHis}$, 15.619kD; SauCas9_PI$_{6XHis}$, 18.206kD; SauCas9_WED-PI$_{6XHis}$, 32.612kD; shAcrIIA13b$_{NoTag}$, 15.248kD). Representative of three experiments (n=3).

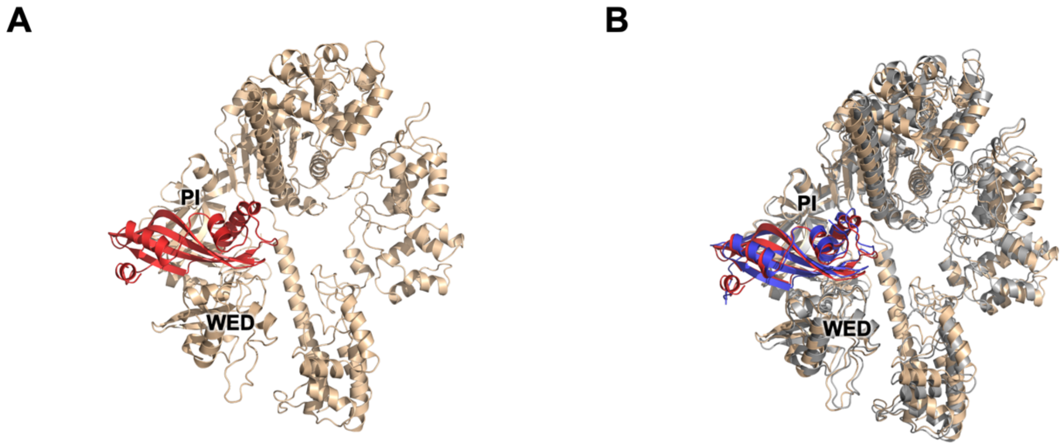

Supplementary Figure 3. Comparison of docking model of *Sau*Cas9 and AcrIIA13b complex generated by HDOCK with that generated by Alphafold2. **(A)** Docking model generated by Alphafold2. **(B)** The docking model of the *Sau*Cas9 and AcrIIA13b complex generated by HDOCK (Gold color) was compared with that generated by Alphafold2 (Gray color). All structural representations were prepared using PyMOL.

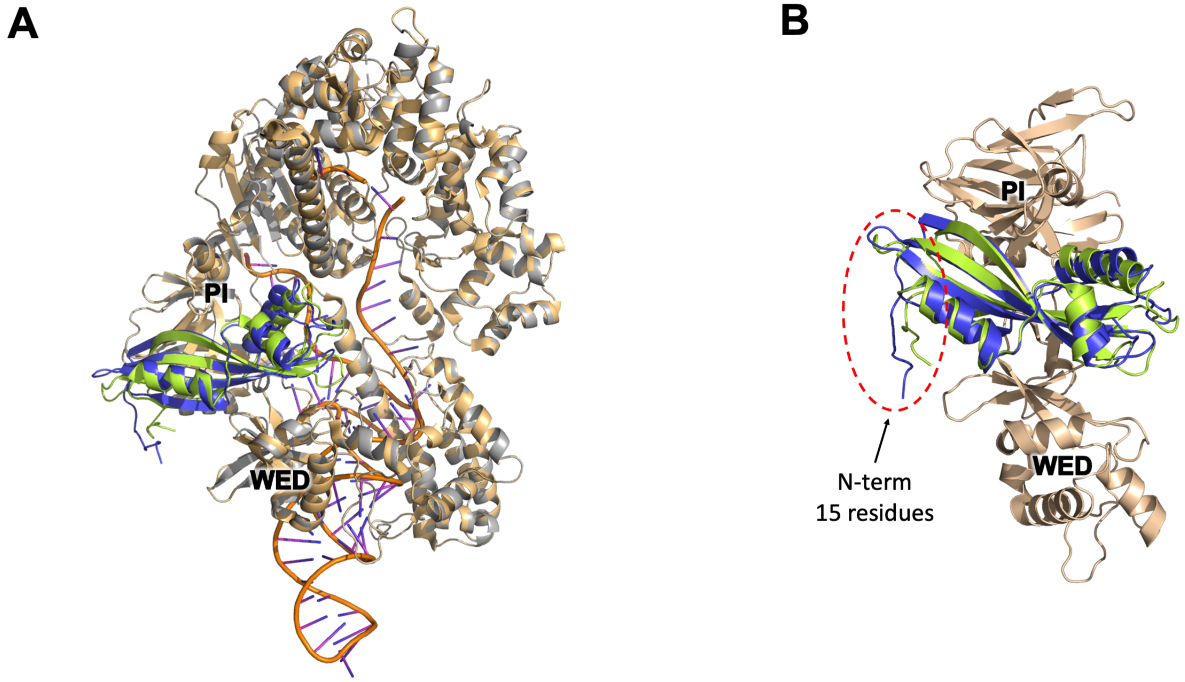

Supplementary Figure 4. Comparison of docking model of *Sau*Cas9-AcrIIA13b complex and crystal structure of SauCas9-AcrIIA13 complex (PDBID: 7ENI).
(A) Superimposition of docking model of SauCas9-AcrIIA13b complex (SauCas9-gray, AcrIIA13b-blue) with SauCas9-AcrIIA13 crystal structure deposited as 7ENI (SauCas9-wheat, AcrIIA13b-limon). **(B)** Closer view of AcrIIA13/AcrIIA13b and WED-PI domain of SauCas9 based on 7ENI structure. 15 residues at N-terminus are indicated with red circle. The structures were visualized using PyMOL.

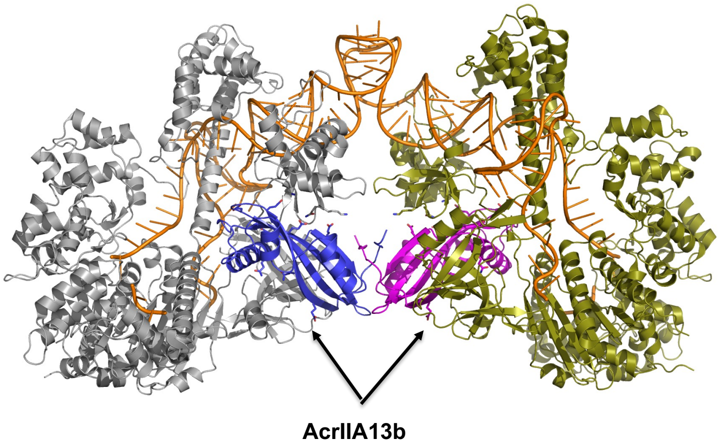

Supplementary Figure 5. Modeled Cas9 dimerization induced by AcrIIA13b dimer. Dimer docking model generated based on the dimeric structure of AcrIIA13b, using the HDOCK server and visualized using PyMOL.

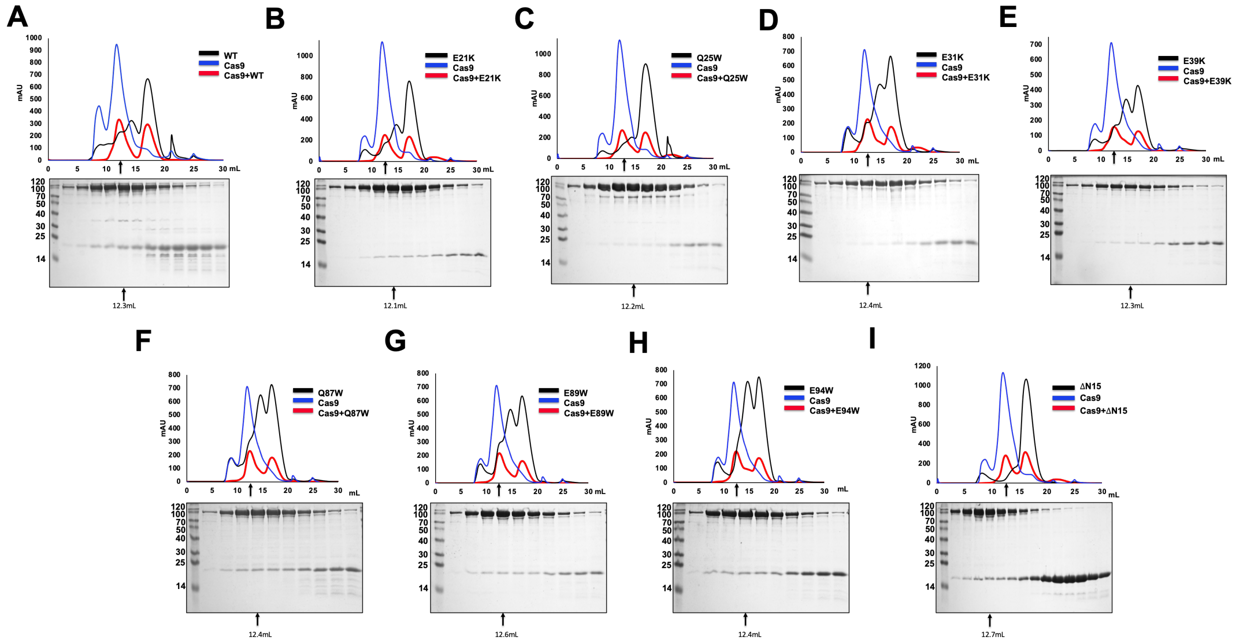

Supplementary Figure 6. Analysis of the interactions between Cas9 and various mutants of AcrIIA13b on SEC followed by SDS-PAGE. (A-I) Interaction analysis of Cas9 with various mutants of AcrIIA13b by Size exclusion chromatography (SEC). SEC profiles produced by the mixture were shown. SDS-PAGE gel produced by main peak and excess AcrIIA13b mutants' peak fractions from the mixture was provided under the SEC profile. Black arrows indicate the fractions eluting around 12ml, which were used for quantification in Fig. 5F and 5G. The elution volumes indicated below the black arrows correspond to each SEC profile. Representative of two experiments (n=2).

A

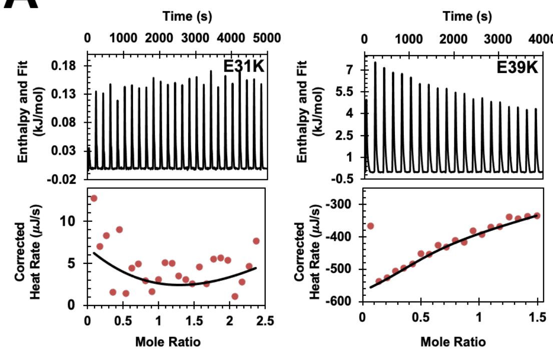

B

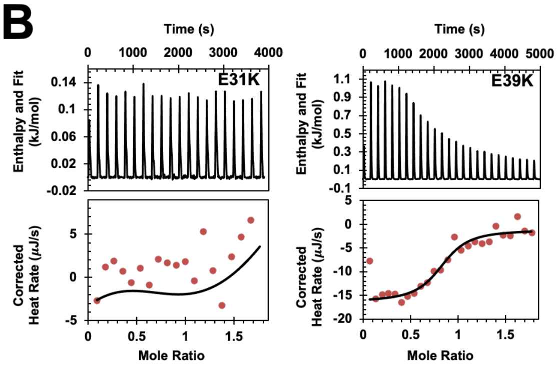

C

Proteins	WT<br>K_d (10^{-6}M)	E31K<br>K_d (10^{-6}M)	E39K<br>K_d (10^{-6}M)
***Sau*Cas9**			
PBS	4.47±0.88	N.D	13.2±2.4
TRIS	6.15±0.85	N.D	33.6±5.6

D

Proteins	WT<br>K_d (10^{-6}M)	E31K<br>K_d (10^{-6}M)	E39K<br>K_d (10^{-6}M)
**WED-PI**			
PBS	0.12±0.02	N.D	1.31±0.8
TRIS	1.61±0.41	N.D	3.36±1.8

Supplementary Figure 7. ITC analysis of AcrIIA13b mutants E31K and E39K binding to SauCas9 and WED-PI domain. (A and B) Isothermal titration calorimetry (ITC) experiments showing titration of two putative protein-protein interface (PPI) disrupting mutants, E31K and E39K, with *Sau*Cas9 **(A)**, and WED-PI **(B)**. ITC was performed at 20 °C in the PBS condition. The raw ITC data are shown in the upper panel, and experimental fitting of the data to a single-site interaction model is shown in the lower panel. **(C and D)** Summary table of ITC experiment results with two mutants of AcrIIA13b (E31K and E39K) and *Sau*Cas9 **(C)** or WED-PI domain **(D)**. Representative of three experiments (n=3).

A B C

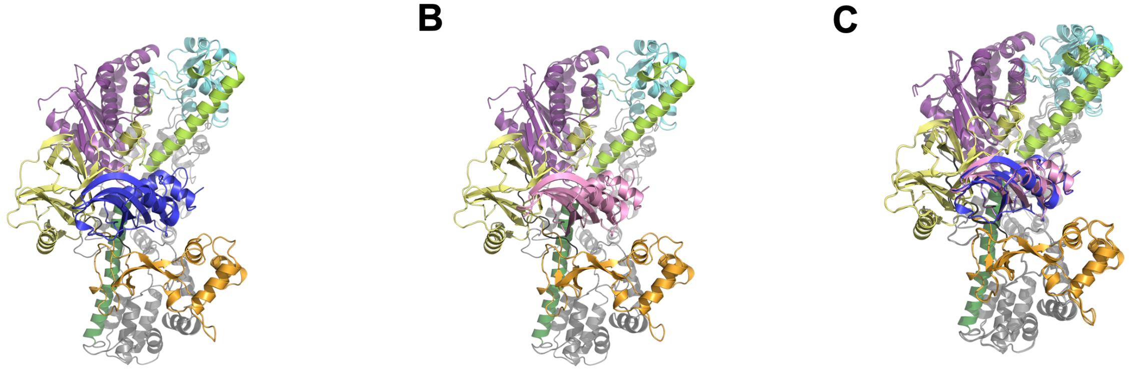

Supplementary Figure 8. Structural docking models of AcrIIA13b WT and ΔN15 mutant with SauCas9 generated by HDOCK server. (A) Complex model of wild-type AcrIIA13b (colored in blue) docked onto SauCas9. **(B)** Complex model of ΔN15 (colored in pink) docked onto SauCas9. **(C)** Structural alignment of the WT and ΔN15 docking models. Docking models were generated by HDOCK server and visualized with PyMOL.

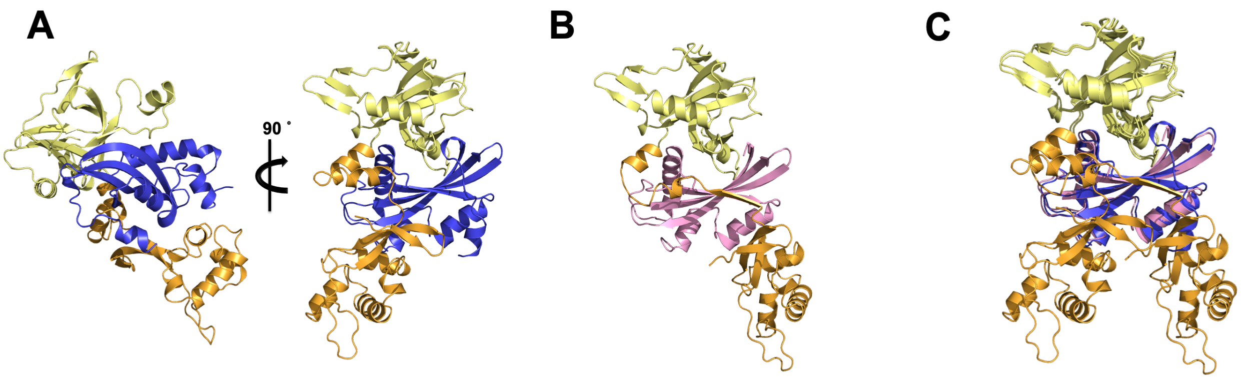

Supplementary Figure 9. AlphaFold3-predicted complex models of AcrIIA13b WT and ΔN15 mutant with the WED-PI domain of SauCas9. (A) Predicted structure of wild-type AcrIIA13b (blue) in complex with the WED-PI domain, shown from two orientations (rotate 90°). **(B)** Predicted complex model of ΔN15 (pink) with WED-PI domain. **(C)** Structural alignment of the WT and ΔN15 complex prediction models, highlighting differences in their binding poses relative to the WED-PI domain (orange and yellow). Complex models were predicted with AlphaFold3 and visualized using PyMOL.

Supplementary Table1. Table summarizing the result of structural similarity search using the Dali server.

Proteins (accession numbers)	Z-score	RMSD (Å)	Identity (%)
**AcrIIA14** (7EL1-E)	5.7	2.7 (78/98)	13
**Surfite reductase** (2XSJ-B)	5.2	5.9 (70/385)	5
**Histone Parylation Factor 1** (6X0L-P)	5.1	3.5 (76/474)	10
**DNA ligase** (6IMJ-A)	4.9	2.9 (71/412)	7
**AcrIIA22** (7JTA-A)	4.8	1.8 (55/56)	10

Supplementary Table2. Oligonucleotides used in this study.

Name	Sequence (5'→3')
AcrIIA13b-F	GGGCATATGAACGAACTGAACAACAAAAT
AcrIIA13b-R	GGGCTCGAGTTCGAAGTAGCCGATTTCCG
delN15(Mut)- F	GGGCATATGGAAGTGGAACTGATCGAAGA
delN15(Mut)- R	GGGCTCGAGTTCGAAGTAGCCGATTTCCG
V12W(Mut)- sense	CTGAACAACAAAATGATTGAAGATTGGGTCCTGGGCGAAGTGGAACTGAT C
V12W(Mut)- antisense	GATCAGTTCCACTTCGCCCAGGACCCAATCTTCAATCATTTTGTTGTTCAG
E21K(Mut)- sense	GCGAAGTGGAACTGATCAAAGATCTGGGCCAGTAC
E21K(Mut)- antisense	GTACTGGCCCAGATCTTTGATCAGTTCCACTTCGC
Q25W(Mut)- sense	ATCGAAGATCTGGGCTGGTACTTCATCGACATCGAA
Q25W(Mut)- antisense	GATGTCGATGAAGTACCAGCCCAGATCTTCGATCAGTT

E31K(Mut)-sense	CCAGTACTTCATCGACATCAAAGGCGATTACGAATACAAC
E31K(Mut)-antisense	GTTGTATTCGTAATCGCCTTTGATGTCGATGAAGTACTGG
E39K(Mut)- sense	GATTACGAATACAACGTGAAATTCGCGACCCTGAGCG
E39K(Mut)- antisense	CGCTCAGGGTCGCGAATTTCACGTTGTATTCGTAATC
Q87W(Mut)- sense	TGGAAAAAGACTGGCAGGAAGAAACCTACATCGAA
Q87W(Mut)- antisense	TTTCTTCCTGCCAGTCTTTTTCCAGCCATTTATCGT
E89W(Mut)- sense	GCTGGAAAAAGACCAGCAGTGGGAAACCTACATCGAAAGCG
E89W(Mut)- antisense	CGCTTTCGATGTAGGTTTCCCACTGCTGGTCTTTTTCCAGC
E94W(Mut)- sense	CAGGAAGAAACCTACATCTGGAGCGTTTTCTTCGTTAAC
E94W(Mut)- antisense	GTTAACGAAGAAAACGCTCCAGATGTAGGTTTCTTCCTG
SauCas9_REC-F	GGGCATATGTACAACCTGCTGACCGACCA
SauCas9_REC-R	GGGCTCGAGTGGTACCAGCTTCAGCCGGT
SauCas9_HNH-F	GGGCATATGCTGATTGAAAAAATCAAGCT
SauCas9_HNH-R	GGGCTCGAGTTCCAGCAGGTACTCCTTTT
SauCas9_WED-F	GGGCATATGCTGTATAGTACAAGAAAAGA
SauCas9_WED-R	GGGCTCGAGCAGTGACAGCTTGACCACCT
SauCas9_PI-F	GGGCATATGAAGCCATACAGATTCGATGT
SauCas9_PI-R	GGGCTCGAGGCCCTTTTTGATAATCTGAG
Target DNA (mIRGM3)	ATGGCGAACTACCTGAAACGTCTGATCAGCCCGTGGTCTAAATCTATGAC<br>CGCGGGTGAATCTCTGTACAGCAGCCAGAACTCTTCTAGCCCGGAAGTTA<br>TTGAAGATATCGGTAAAGCGGTTACCGAAGGCAATCTGCAGAAAGTTATC<br>GGTATCGTTAAAGATGAAATTCAGTCTAAATCTCGTTACCGTGTGAAAATT<br>GCGGTTACCGGCGATTCTGGTAACGGCATGAGCTCCTTCATCAACGCACT<br>GCGTTTCATCGGTCACGAAGAAGAAGATTCTGCGCCGACCGGTGTTGTTC<br>GTACCACCAAAAAACCGGCATGCTACAGCTCTGATAGCCACTTCCCGTAC<br>GTTGAACTGTGGGACCTGCCGGGTCTGGGCGCTACCGCTCAGTCTGTTG<br>AATCTTACCTGGAAGAAATGCAGATTAGCACCTTCGATCTGATCATTATCG<br>TTGCGTCTGAACAGTTTAGCTCTAACCACGTTAAACTGGCGATCACCATG<br>CAGCGTATGCGTAAACGTTTCTATGTTGTTTGGACTAAACTGGATCGCGAT<br>CTGTCTACTTCTACTTTCCCGGAACCGCAGCTGCTGCAGTCCATCCAGCG<br>TAACATTCGCGAAAACCTGCAGCAGGCTCAGGTTCGTGACCCGCCGCTG<br>TTCCTGATCAGCTGCTTCAGCCCGTCCTTCCACGATTTCCCGGAACTGCG<br>TAACACCCTGCAGAAAGACATCTTCAGCATCCGTTATCGTGATCCGCTGG<br>AAATCATCTCTCAGGTTTGTGATAAATGCATTAGCAACAAAGCCTTCAGCC<br>TGAAAGAAGATCAGATGCTGATGAAAGATCTGGAAGCAGCAGTTAGCTCT<br>GAAGATGATACCGCGAACCTGGAACGTGGCCTGCAGACCTATCAGAAACT<br>GTTTGGTGTTGATGATGGTTCTCTGCAGCAGGTTGCGCGTTCTACCGGTC<br>GTCTGGAAATGGGCAGCCGTGCGCTGCAGTTCCAGGACCTGATTAAAAT<br>GGATCGTCGTCTGGAACTGATGATGTGCTTCGCGGTTAACAAATTCCTGC<br>GCCTGCTGGAAAGCTCTTGGTGGTATGGCCTGTGGAACGTTGTTACCCGT<br>TACTTCCGTCACCAGCGTCACAAACTGGTTATCGAAATCGTTGCGGAAAA<br>CACCAAAACCAGCCTGCGTAAAGCGCTGAAAGATA
Target oligo-sense<br>(for sgRNA synthesis)	TTCTAATACGACTCACTATAGCAGCTGATCAGGAACAGCGGTTTTAGTACT<br>CTGGAAACAGAATCTACTAAAACAAGGCAAAATGCCGTGTTTATCTCGTCA<br>ACTTGTTGGCGAGATTT
Target oligo-antisense<br>(for sgRNA synthesis)	AAATCTCGCCAACAAGTTGACGAGATAAACACGGCATTTTGCCTTGTTTTA<br>GTAGATTCTGTTTCCAGAGTACTAAAACCGCTGTTCCTGATCAGCTGCTAT<br>AGTGAGTCGTATTAGAA
SauCas9sgRNA	GCAGCUGAUCAGGAACAGCGGUUUUAGUACUCUGGAAACAGAAUCUAC<br>UAAAACAAGGCAAAAUGCCGUGUUUAUCUCGUCAACUUGUUGGCGAGA<br>UUU
